# A contextual genomic perspective on physical activity and its relationship to health, well being and illness

**DOI:** 10.1038/s41588-025-02260-9

**Published:** 2025-07-21

**Authors:** Marco Galimberti, Daniel F. Levey, Joseph D. Deak, Keyrun Adhikari, Cassie Overstreet, Priya Gupta, Rachana Nitin, Hang Zhou, Nicole J. Lake, Kelly M. Harrington, Luc Djousse, Lea K. Davis, VA Million Veteran Program, Marco Galimberti, Daniel F. Levey, Joseph D. Deak, Keyrun Adhikari, Cassie Overstreet, Priya Gupta, Hang Zhou, Kelly M. Harrington, Luc Djousse, J. Michael Gaziano, Murray B. Stein, Joel Gelernter, J. Michael Gaziano, Murray B. Stein, Joel Gelernter

**Affiliations:** 1 Veterans Affairs Connecticut Healthcare System, West Haven, CT USA; 2 Department of Psychiatry, Yale School of Medicine, New Haven, CT USA; 3 Department of Medicine, Vanderbilt University Medical Center, Nashville, TN USA; 4 Department of Genetics, Yale School of Medicine, New Haven, CT USA; 5 Department of Psychiatry, Boston University School of Medicine, Boston, MA USA; 6 Million Veteran Program (MVP) Coordinating Center, VA Boston Healthcare System, Boston, MA USA; 7 Department of Medicine, Brigham and Women’s Hospital, Boston, MA USA; 8 Department of Medicine, Harvard Medical School, Boston, MA USA; 9 VA San Diego Healthcare System, San Diego, CA USA; 10 Department of Psychiatry and School of Public Health, University of California, San Diego, La Jolla, CA USA

**Keywords:** Genome-wide association studies, Disease prevention

## Abstract

Physical activity (PA) is one of the most fundamental traits in the animal kingdom, has pervasive health benefits, and is genetically influenced. Using data from the Million Veteran Program, we conducted genetic analyses of leisure, work and home-time PA. For leisure, we included 189,812 individuals of European ancestry (SNP-based heritability (*h*^2^) = 0.083 ± 0.005), 27,044 of African ancestry (*h*^2^ = 0.034 ± 0.017) and 10,263 of Latin American ancestry (*h*^2^ = 0.083 ± 0.036) in a cross-ancestry meta-analysis with UK Biobank data, identifying 70 lead variants. Leisure-time PA was genetically distinct from PA at home or work, with the latter two showing less health benefit with respect to health outcomes and lifespan. Mendelian randomization analyses showed a protective role of leisure-time PA against COVID-19 hospitalization (*β* = −0.067 ± 0.016; *P* = 2.8 × 10^−^^5^), and with other traits including cardiovascular and respiratory system diseases, metabolic traits and aging. These findings provide new insights into the biology of PA, showing specific causal health benefits of leisure-time PA.

## Abstract (web summary)

Genome-wide association meta-analyses of leisure, work and home-time physical activity (PA) from European, African and Latin American ancestries provide insights into the biology of PA and its relationship to health and diseases.

## Main

Physical activity (PA) has pervasive health benefits, including beneficial influences on chronic conditions like cancer, hypertension and type 2 diabetes^1^. The majority of these associations are supported by cross-sectional studies^2–5^, which have great utility but cannot establish causality or determine the direction of causal relationships.

An individual’s level of PA is influenced by genetic factors, with estimated heritability (*h*^2^) between 48% and 71%^6^. To identify genetic variants relevant to PA, genome-wide association studies (GWAS) have been conducted, initially focusing on PA during leisure time^7–10^. Identifying genetic associations with PA affords us a unique opportunity; because we are born with genetic variations before exposures to psychological, social and environmental factors that may affect activity levels, we can begin to decompose some of the complex interactions that underlie and potentially confound the study of this important health trait. There is also a wide range of interindividual differences in requirements for PA in daily life. Some jobs require much more physical exertion than others; some individuals must exert more effort in their households than others. These kinds of PA tend to differ phenomenologically from exercise taken at leisure, occurring more in concentrated bursts in the first two cases, and with the potential to be more sustained in the latter case; yet the level of activity in different contexts is often treated as if equivalent. There are, however, prior reports that there may not be comparable health benefits for activity at leisure versus activity in other contexts^11,12^, and genetic analyses may help shed light on such differences.

Two previous studies of PA used UK Biobank (UKB) data^13,14^. The first study (*n* = 91,105) identified three variants associated with PA, based on wrist-worn accelerometer data^14^. The second study (*n* ≤ 377,234) identified ten significant loci across five phenotypes, three based on self-report data (moderate-to-vigorous PA (MVPA), vigorous PA (VPA) and strenuous sport or other exercise (SSOE) during leisure time), and two on wrist-worn accelerometer data^13^. A multiancestry meta-analysis combining data from 703,901 individuals from 51 studies identified 11 associated loci for MVPA^15^. Combining this phenotype with two traits phenotypically contrary to PA (leisure screen time and sedentary behavior at work), 99 associated loci were found, summing results from PA (11 loci), leisure screen time (88 loci) and sedentary behavior at work (4 loci); that is, most loci were mapped for phenotypes correlated to a lack of PA^15^.

We sought to maximize power to investigate PA genetics using a quantitative trait definition, combining different intensities of PA by incorporating their frequencies, using data from the Million Veteran Program (MVP). We focused on PA per se, as opposed to sedentary behavior, with our primary quantitative trait, All-PA–leisure, focused exclusively on PA during leisure time (Methods) with data from European (EUR; *n* = 189,812), African (AFR; *n* = 27,044) and Latin American (AMR; *n* = 10,263) ancestries. We studied this trait in two meta-analyses (All-PA–leisure + SSOE), one of EUR ancestry (*n* = 511,310) and one cross-ancestry (EUR, AFR and AMR ancestries; *n* = 548,617), with SSOE data from UKB (*n*_eff_ = 4/(1/*n*_cases_ + 1/*n*_controls_) = 321,498), also based on information regarding PA during leisure time (Supplementary Fig. 1 and Supplementary Table 1)^13^. In doing so, we substantially increased the number of discovered loci while also enhancing the diversity of the sample beyond EUR. This enabled us to investigate, from a genomic perspective, the relationship of PA to a range of health traits, and especially protective effects of PA against health outcomes including type 2 diabetes, gastroesophageal reflux disease, abdominal aortic aneurysm, heart failure, hospitalization caused by COVID-19, osteoarthritis, parental survival and an aging phenotype. We also investigated PA traits related to different activity contexts (home, work, and leisure), for which we found substantial genetic differences.

## Results

### GWAS of All-PA–leisure and All-PA–leisure + SSOE meta-analysis

#### All-PA–leisure and SSOE phenotypes

GWAS of All-PA–leisure in MVP EUR data identified 14 lead SNPs at 13 loci with a genome-wide significant (GWS) threshold *P* value of 5 × 10^−8^ (or 6 lead SNPs in 6 loci if considering a threshold *P* = 5 × 10^−9^; Supplementary Fig. 2 and Supplementary Table 2), whereas there were no significant GWAS findings for MVP AFR and AMR data. GWAS of SSOE in UKB identified 15 lead SNPs in 14 loci with a threshold *P* value of 5 × 10^−8^ (8 lead SNPs in 7 loci if considering a threshold *P* *=* 5 × 10^−9^; Supplementary Fig. 3 and Supplementary Table 3).

The two most significant lead SNPs in the GWAS for All-PA–leisure (EUR, rs761898, *P* = 1.63 × 10^−11^; rs7613360, *P* = 3.15 × 10^−11^) map to intergenic regions on chromosomes 14 and 3, respectively (Supplementary Table 3). Additional GWS loci included SLC39A8*rs13107325 (*P* = 1.57 × 10^−10^; solute carrier family 39 member 8). As previously described^13^, the most significant variant for GWAS of SSOE in UKB is CADM2*rs62253088 (*P* = 1.0 × 10^−19^ in this study; *Cell Adhesion Molecule 2*).

A sex-stratified analysis of the X chromosome in MVP (All-PA–leisure) showed no GWS variants (Supplementary Fig. 4).

#### All-PA–leisure + SSOE meta-analyses

We calculated SNP-based heritability (SNP-*h*^2^) in MVP data for All-PA–leisure (Table 1), and the genetic correlation between All-PA–leisure and traits from the previous UKB report^13^ (Supplementary Notes).

**Table 1:** Estimated SNP-heritability (*h*^2^) of the cohorts used in the study

Database	Ancestry	Phenotype	*h*^2^	s.e.	*Z* score	*P* value	*n*
MVP	EUR	All-PA–leisure	0.083	0.005	17.57	4.2 × 10^−69^	189,812
UKB	EUR	SSOE	0.061	0.003	20.33	7.0 × 10^−92^	321,498
MVP	AFR	All-PA–leisure	0.034	0.017	2.0	0.046	27,044
MVP	AMR	All-PA–leisure	0.083	0.036	2.31	0.021	10,263
MVP + UKB	EUR	All-PA–leisure + SSOE	0.058	0.002	29	6.6 × 10^−185^	511,310

The *n* represents sample size (the effective sample size 4/(1/*n*_cases_ + 1/*n*_controls_) for the SSOE phenotype). Tests were one-sided.

**Fig. 1:**
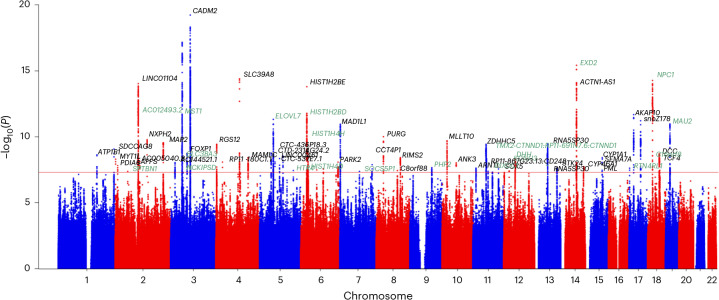
Cross-ancestry All-PA–leisure + SSOE meta-analysis. Annotated genes are the closest to each significant lead SNP. Different genes compared to EUR All-PA–leisure meta-analysis (Supplementary Fig. 6) are highlighted in green. The red horizontal line indicates GWS (*P* = 5 × 10^−8^) level, where *P* values were calculated using sample-size-based approach, two-sided tests.

The EUR All-PA–leisure + SSOE meta-analysis, including the UKB phenotype SSOE and the MVP phenotype All-PA–leisure in a sample-size-based meta-analysis, greatly increased discovery, yielding 67 lead SNPs in 55 loci (Supplementary Fig. 5 and Supplementary Table 4), with intercept of 1.038 ± 0.011 and an attenuation ratio of the linkage disequilibrium score regression (LDSC) of 0.057 ± 0.016 (ref. ^16^). The cross-ancestry meta-analysis provided 70 lead SNPs in 56 loci (Fig. 1 and Supplementary Table 5). Differences in the number of lead SNPs and associated loci considering a more stringent threshold *P* ≤ 5 × 10^−^^9^ are reported in Supplementary Table 6. In both the meta-analyses, the strongest association was with CADM2*rs62253088 (EUR meta-analysis *P* = 2.0 × 10^−21^; cross-ancestry meta-analysis *P* = 6.1 × 10^−20^). The second strongest associated lead SNP was also on chromosome 3: MST1R*rs3733134 (EUR meta-analysis *P* = 1.4 × 10^−17^; cross-ancestry meta-analysis *P* = 6.9 × 10^−18^).

#### Gene-based and gene-set analyses of All-PA–leisure + SSOE

Focusing on the EUR meta-analysis, the gene-based test using MAGMA identified 119 significant genes from 19,030 protein-coding genes (GWS defined at *P* = 2.6 × 10^−6^; Supplementary Table 7). *CADM2* was the most significant gene (*P* = 3.7 × 10^−25^) followed by *NPC1* (*P* = 4.8 × 10^−16^). The fastENLOC^17,18^ analysis identified 41 genes with gene-level colocalization probability (GLCP) ≥ 0.9 (Supplementary Table 8).

For the EUR All-PA–leisure + SSOE meta-analysis, we also conducted transcriptome-wide association study (TWAS) and fine-mapping (Supplementary Results; Supplementary Tables 9–11). These analyses, together with the previously mentioned gene-based approaches, highlighted two genes (*CADM2* and *AKAP10*) shared by four approaches, and eight genes (*RGS12*, *NCP1*, *GATAD2A*, *DLAT*, *CYP46A1*, *ANK3, AFF3 and ACTN1*) shared by three approaches (discussion in ‘Supplementary Results—Genes detected by gene-based approaches’). We also conducted MTAG analysis with leisure screen time and liking of PA (‘Supplementary Results—MTAG analyses of EUR All-PA–leisure + SSOE’; Supplementary Tables 12 and 13).

The following four significant gene ontology (GO) terms resulted from the gene-set analysis: structural constituent of the presynapse, presynaptic active zone organization, maintenance of presynaptic active zone structure and structural constituent of the synapse (Supplementary Table 14).

#### Tissue and cell-type specificity of All-PA–leisure + SSOE

Tissue Expression Analysis showed 11 significant enrichments in brain tissues, with the two most significant represented in the cerebellar hemisphere and in the cerebellum (Supplementary Figs. 6 and 7). For gene-based association analyses and gene-set analyses, similar results were obtained for the cross-ancestry meta-analysis (Supplementary Notes; Supplementary Tables 15 and 16 and Supplementary Figs. 8 and 9).

Cell-type-enrichment analysis was performed with a collection of human cell types from brain, blood and pancreas (all available tissues). In the EUR meta-analysis of All-PA–leisure + SSOE, the only significant cell type was *GABA*^*+*^ neurons from the GSE76381 Linnarsson Human Midbrain dataset (adjusted *P* value per dataset after multiple testing correction = 2.4 × 10^−^^5^). In the cross-ancestry meta-analysis, no cell types were significantly enriched. The results likely reflect the limited number of kinds of tissue included in this analysis.

#### Functional enrichment analysis

Functional enrichment analysis with g:Profiler using significant genes from the gene-based test as input, identified three significant GO terms shared by the EUR meta-analysis and the cross-ancestry meta-analysis (Supplementary Tables 17 and 18)—presynaptic active zone cytoplasmic component, cell cortex region and synapse. A human phenotype ontology term, polyclonal elevation of IgM, was also shared by the EUR meta-analysis and cross-ancestry meta-analysis.

#### Genetic correlations

**Fig. 2:**
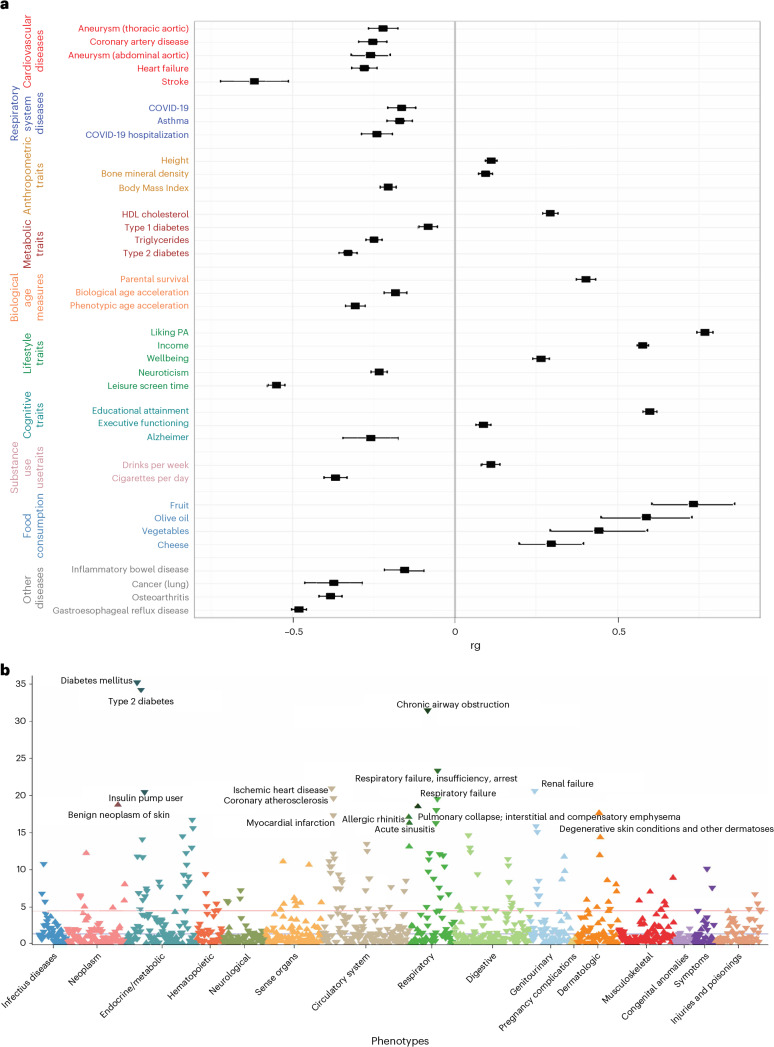
Traits genetically correlated with All-PA–leisure + SSOE. **a**, Genetic correlations of EUR meta-analysis for All-PA–leisure + SSOE phenotype with traits of interest. We display here the traits of interest that were significant after the Benjamini–Hochberg false discovery procedure. Tests were two-sided. Black error bars represent the standard error. **b**, PheWAS of PA from the polygenic score derived from the EUR All-PA–leisure + SSOE meta-analysis. The red line represents the Bonferroni threshold *P* = 3.99 × 10^−5^. Up-pointing and down-pointing triangles represent positive or negative associations, respectively, which were calculated using a logistic regression model.

We estimated the genetic correlation of the All-PA–leisure + SSOE phenotype (EUR meta-analysis) with traits of interest covering a broad range of diseases and physiological traits, using the Benjamini–Hochberg false discovery rate correction (Methods). Results showed significant negative genetic correlations between All-PA–leisure + SSOE with all disease traits studied, with the strongest values for stroke (*r*_*g*_ = −0.62 ± 0.11; *P* = 3.54 × 10^−9^) and gastroesophageal reflux disease (*r*_*g*_ = −0.482 ± 0023; *P* = 9.9 × 10^−97^; Fig. 2a, Supplementary Table 19 and Supplementary Notes). Conditional analysis adjusting for income as a measure of socioeconomic status (Supplementary Fig. 10), highlighted a few changes compared to previous genetic correlations (Supplementary Table 20). Genetic correlations of All-PA–leisure + SSOE with height and executive functioning became nonsignificant, whereas multiple sclerosis became significant (*r*_*g*_ = −0.184 ± 0.086; *P* = 0.032). Consistent results were obtained from a phenome-wide association analysis (PheWAS; Fig. 2b; Supplementary Notes; Supplementary Table 21).

#### Local genetic correlations

We next considered local correlations (LAVA^19^) as global genetic correlations may mask important regional differences. There were 91 significant local bivariate genetic correlations at 61 loci between the EUR All-PA–leisure + SSOE meta-analysis and 18 traits in the genetic correlation analyses (Supplementary Table 21). Of these, oral cancer was not flagged by the global genetic correlation analysis. The trait with the highest number of significant local genetic correlations with All-PA–leisure + SSOE was educational attainment (31 loci), followed by body mass index (BMI; 15 loci) and income (9 loci). For two traits, height and well being, we observed local genetic correlations with EUR All-PA–leisure+SSOE in opposite directions (Supplementary Table 22).

#### Mendelian randomization (MR) analyses of All-PA–leisure

We used MR analyses to investigate the causal relationship between All-PA–leisure and traits with which it had a significant genetic correlation. Since several statistics for target traits included UKB data, we used the GWAS of All-PA–leisure in EUR MVP (and not the meta-analysis) to avoid sample overlap. The following four methods were tested: MR-Egger, weighted median, inverse-variance weighted (IVW) and simple mode. Using a threshold *P* < 1 × 10^−5^ for instrumental variables, we observed significant bidirectional negative (protective) effects of All-PA–leisure with respect to BMI, type 2 diabetes, leisure screen time, cigarettes per day and gastroesophageal reflux disease (Tables 2 and 3). Unlike bidirectional effects, which show effects both caused by All-PA–leisure and on All-PA–leisure (that is, PA increases or decreases risk for the comparator trait, and that trait also increases or decreases PA), unidirectional effects show only one direction of causality. There were unidirectional negative (protective) effects of All-PA–leisure on abdominal aortic aneurysm, heart failure, COVID-19 hospitalization, triglycerides, phenotypic age acceleration (an age-adjusted biological age measure; Supplementary Methods) and osteoarthritis (Tables 2 and 3). We found significant bidirectional positive effects between All-PA–leisure and HDL-cholesterol (HDL-C), parental survival, liking of PA, household income, well being and educational attainment. MR analyses with a stronger threshold *P* = 5 × 10^−8^ for instrumental variables showed similar causal relationships, and also a possible protective effect of All-PA–leisure on asthma (Supplementary Notes; Supplementary Tables 23–25).

**Table 2:** MR analysis of causal effects of PA during leisure time (All-PA–leisure) on traits of interest

Outcome	*n*	IVW		MR-Egger		Weighted median		Simple mode	
		*β* (s.e.)	*P* value	*β* (s.e.)	*P* value	*β* (s.e.)	*P* value	*β* (s.e.)	*P* value
Aneurysm (thoracic aortic)	70	−0.031 (0.020)	0.12	0.036 (0.071)	0.61	−0.025 (0.027)	0.35	−0.021 (0.064)	0.74
Coronary artery disease	53	−0.04 (0.02)	0.034	−0.01 (0.10)	0.89	−0.03 (0.02)	0.14	−0.03 (0.05)	0.51
Aneurysm (abdominal aortic)	70	**−0.119 (0.028)**	**2.2** **×** **10**^**−5**^	0.02 (0.10)	0.81	−0.089 (0.041)	0.029	−0.043 (0.096)	0.64
Heart failure	72	**−0.040 (0.009)**	**6** **×** **10**^**−6**^	−0.04 (0.03)	0.23	−0.03 (0.01)	5 × 10^−3^	−0.02 (0.03)	0.50
Stroke	72	−8.0 × 10^−4^ (3.4 × 10^−4^)	0.017	−1.3 × 10^−3^ (1.2 × 10^−3^)	0.30	−4.6×10^−4^ (4.2 × 10^−4^)	0.27	0.2 × 10^−3^ (1.3 × 10^−3^)	0.89
COVID-19	72	−0.015 (0.005)	5.3×10^−3^	0.003 (0.021)	0.87	−0.007 (0.006)	0.27	−0.003 (0.017)	0.84
Asthma	72	−0.005 (0.002)	0.006	−0.008 (0.006)	0.23	−0.005 (0.002)	0.017	−0.007 (0.006)	0.23
COVID-19 hospitalization	71	**−0.047 (0.011)**	**3.3** **×** **10**^**−5**^	−0.057 (0.043)	0.18	−0.043 (0.013)	1.4 × 10^−3^	−0.045 (0.035)	0.21
Height	72	0.0079 (0.0062)	0.21	0.039 (0.022)	0.086	**0.0108 (0.0026)**	**4.1** **×** **10**^**−5**^	0.0134 (0.0056)	0.019
Bone mineral density	73	0.0044 (0.0051)	0.39	0.003 (0.018)	0.86	0.0094 (0.0049)	0.055	0.012 (0.013)	0.36
BMI	53	**−0.028 (0.007)**	**2.9** **×** **10**^**−5**^	−0.08 (0.03)	0.02	−0.020 (0.006)	1.9 × 10^−3^	−0.018 (0.015)	0.23
HDL-C	72	**0.0111 (0.0023)**	**2.5** **×** **10**^**−6**^	0.0135 (0.0085)	0.12	**0.0069 (0.0014)**	**1.6** **×** **10**^**−6**^	0.0070 (0.0034)	0.045
Type 1 diabetes	73	−0.026 (0.018)	0.14	−0.046 (0.065)	0.49	−0.043 (0.022)	0.048	−0.082 (0.056)	0.15
Triglycerides	72	**−0.0194 (0.0042)**	**3.5×10**^**−6**^	−0.002 (0.015)	0.88	**−0.0150 (0.0037)**	**4.4** **×** **10**^**−5**^	−0.0170 (0.0094)	0.073
Type 2 diabetes	53	**−0.068 (0.016)**	**1.6** **×** **10**^**−5**^	−0.087 (0.084)	0.31	**−0.061 (0.015)**	**4.0** **×** **10**^**−5**^	−0.078 (0.042)	0.072
Parental survival	67	**−0.0287 (0.0051)**	**2.4** **×** **10**^**−8**^	0.009 (0.019)	0.63	**0.0208 (0.0061)**	**5.8** **×** **10**^**−4**^	0.017 (0.015)	0.26
Biological age acceleration	72	−0.0121 (0.0094)	0.20	−0.021 (0.034)	0.54	−0.016 (0.012)	0.16	−0.014 (0.025)	0.57
Phenotypic age acceleration	72	**−0.112 (0.028)**	**6.5** **×** **10**^**−5**^	0.02 (0.10)	0.88	−0.070 (0.030)	0.022	−0.046 (0.079)	0.56
Liking of PA	80	**0.0341 (0.0047)**	**2.9** **×** **10**^**−13**^	0.003 (0.017)	0.86	**0.0302 (0.0052)**	**6.9** **×** **10**^**−9**^	0.042 (0.013)	2.0×10^−3^
Income	72	**0.038 (0.005)**	**7** **×** **10**^**−16**^	0.002 (0.017)	0.90	**0.030 (0.004)**	**2** **×** **10**^**−11**^	0.017 (0.013)	0.17
Well being	62	**7.9** **×** **10**^**−3**^ **(2.2** **×** **10**^**−3**^**)**	**3.7** **×** **10**^**−4**^	−7.6×10^−3^ (9.5×10^−3^)	0.43	3.3×10^−3^ (1.7 × 10^−3^)	0.056	1.7 × 10^−3^ (5.0 × 10^−3^)	0.74
Neuroticism	72	−0.014 (0.005)	0.004	0.022 (0.018)	0.21	−7.6×10^−3^ (4.4 × 10^−3^)	0.09	−0.015 (0.010)	0.15
Leisure screen time	73	**−0.0565 (0.0075)**	**7.0** **×** **10**^**−14**^	−0.016 (0.027)	0.55	**−0.0366 (0.0066)**	**2.3** **×** **10**^**−8**^	−0.034 (0.019)	0.082
Educational attainment	69	**0.0496 (0.0052)**	**2** **×** **10**^**−21**^	0.003 (0.020)	0.86	**0.0272 (0.0035)**	**7.6** **×** **10**^**−15**^	0.0248 (0.0084)	4.4 × 10^−3^
Executive functioning	69	0.0015 (0.0018)	0.39	−0.0019 (0.0074)	0.79	0.0006 (0.0018)	0.75	−0.0083 (0.0055)	0.13
Alzheimer	73	−0.003 (0.002)	0.11	−0.004 (0.008)	0.65	−0.003 (0.003)	0.37	−0.001 (0.007)	0.84
Drinks per week	70	8.2 × 10^−3^ (3.3 × 10^−3^)	0.014	0.014 (0.013)	0.28	5.1×10^−3^ (3.0 × 10^−3^)	0.090	6.6 × 10^−3^ (8.6 × 10^−3^)	0.44
Cigarettes per day	70	**−4.07** **×** **10**^**−2**^ **(0.78** **×** **10**^**−2**^**)**	**1.77** **×** **10**^**−5**^	0.010 (0.029)	0.73	**−4.17** **×** **10**^**−2**^ **(0.88** **×** **10**^**−2**^**)**	**1.98** **×** **10**^**−6**^	−0.068 (0.027)	0.015
Fruit	72	**0.0118 (0.0024)**	**1.07** **×** **10**^**−6**^	0.0040 (0.0087)	0.65	0.0089 (0.0034)	9.95 × 10^−3^	0.0033 (0.0091)	0.72
Olive oil	72	0.0077 (0.0033)	0.019	0.031 (0.012)	8.80E−03	0.0092 (0.0042)	0.03	0.011 (0.012)	0.34
Vegetables	72	0.0010 (0.0022)	0.66	−0.0141 (0.0079)	0.078	−0.0006 (0.0031)	0.84	0.0011 (0.0080)	0.89
Cheese	72	0.0028 (0.0032)	0.39	−0.004 (0.012)	0.74	0.0024 (0.0043)	0.58	0.002 (0.011)	0.83
Inflammatory bowel disease	73	−0.0019 (0.0012)	0.10	−0.0002 (0.0029)	0.95	−0.0004 (0.0017)	0.80	−0.0017 (0.0033)	0.60
Cancer (lung)	73	−0.044 (0.034)	0.19	−0.26 (0.12)	0.042	0.016 (0.043)	0.71	0.08 (0.11)	0.50
Osteoarthritis	72	**−0.0036 (0.0008)**	**1.15** **×** **10**^**−5**^	−0.0032 (0.0030)	0.29	−0.0026 (0.0009)	5.3 × 10^−3^	−0.0026 (0.0023)	0.27
Gastroesophageal reflux disease	70	**−0.063 (0.011)**	**5.8** **×** **10**^**−9**^	0.009 (0.040)	0.83	**−0.058 (0.010)**	**1.5** **×** **10**^**−9**^	−0.070 (0.031)	2.6 × 10^−2^

Significant results after multiple testing correction (*P* < 6.9 × 10^−4^) are highlighted in bold. Tests were two-sided.

**Table 3:** MR analysis of causal effects of traits of interest on PA during leisure time (All-PA–leisure)

Exposure	*n*	IVW		MR-Egger		Weighted Median		Simple Mode	
		*β* (s.e.)	*P* value	*β* (s.e.)	*P* value	*β* (s.e.)	*P* value	*β* (s.e.)	*P* value
Aneurysm (thoracic aortic)	30	−0.005 (0.056)	0.93	0.11 (0.14)	0.43	0.117 (0.068)	0.086	0.11 (0.13)	0.41
Coronary artery disease	21	−0.01 (0.07)	0.85	−0.03 (0.13)	0.85	−0.07 (0.07)	0.31	−0.1 (0.1)	0.51
Aneurysm (abdominal aortic)	23	0.029 (0.051)	0.57	−0.02 (0.13)	0.91	0.042 (0.062)	0.50	0.06 (0.13)	0.67
Heart failure	48	−0.2 (0.1)	0.07	0.8 (0.3)	7 × 10^−3^	0.0 (0.1)	0.99	0.1 (0.2)	0.74
Stroke	22	−0.5 (4.4)	0.90	−8.4 (8.4)	0.33	5.1 (6.0)	0.39	7.6 (11.4)	0.51
COVID-19	52	−0.22 (0.15)	0.14	−0.09 (0.27)	0.73	−0.13 (0.20)	0.49	−0.74 (0.39)	0.07
Asthma	44	−0.3 (0.5)	0.55	−1.6 (1.5)	0.32	−1.1 (0.7)	0.10	−0.8 (1.2)	0.50
COVID-19 (hospitalized)	85	−0.04 (0.05)	0.48	0.01 (0.10)	0.96	−0.02 (0.08)	0.80	0.12 (0.17)	0.48
Height	722	0.208 (0.078)	8.0 × 10^−3^	−0.12 (0.16)	0.44	0.14 (0.10)	0.17	0.36 (0.28)	0.19
Bone mineral density	366	−0.046 (0.063)	0.46	−0.10 (0.13)	0.44	−0.050 (0.091)	0.59	−0.02 (0.23)	0.93
BMI	141	**−1.02 (0.17)**	**3.3** **×** **10**^**−9**^	−0.18 (0.48)	0.73	**−0.82 (0.19)**	**1.7** **×** **10**^**−5**^	−1.63 (0.49)	1.1 × 10^−3^
HDL-C	360	**0.77 (0.22)**	**4.4** **×** **10**^**−4**^	−0.32 (0.33)	0.33	0.18 (0.30)	0.55	−0.03 (0.72)	0.97
Type 1 diabetes	153	−0.004 (0.018)	0.81	−0.003 (0.025)	0.91	−0.018 (0.022)	0.41	0.011 (0.069)	0.87
Triglycerides	297	−0.262 (0.091)	4.1×10^−3^	0.22 (0.13)	0.094	−0.09 (0.13)	0.45	0.13 (0.29)	0.66
Type 2 diabetes	210	**−0.156 (0.044)**	**3.7** **×** **10**^**−4**^	−0.073 (0.066)	0.27	0.09 (0.10)	0.38	0.01 (0.15)	0.94
Parental survival	86	**0.67 (0.17)**	**9.6** **×** **10**^**−5**^	−0.32 (0.39)	0.41	0.64 (0.22)	3.1 × 10^−3^	1.42 (0.52)	7.8 × 10^−3^
Biological age acceleration	76	−0.100 (0.071)	0.16	0.10 (0.16)	0.51	0.042 (0.096)	0.66	−0.06 (0.24)	0.79
Phenotypic age acceleration	116	−0.068 (0.025)	7.0 × 10^−3^	−0.078 (0.070)	0.27	−0.037 (0.030)	0.21	0.029 (0.090)	0.75
Liking of PA	46	**1.42 (0.33)**	**2.2** **×** **10**^**−5**^	−0.8 (1.2)	0.51	**1.51 (0.35)**	**1.5** **×** **10**^**−5**^	1.88 (0.85)	0.031
Income	177	**2.12 (0.19)**	**4** **×** **10**^**−28**^	0.85 (0.66)	0.20	**1.55 (0.22)**	**4** **×** **10**^**−12**^	1.27 (0.79)	0.11
Well being	276	**2.4 (0.4)**	**4** **×** **10**^**−9**^	**6.6 (1.8)**	**3** **×** **10**^**−4**^	**2.0 (0.5)**	**2** **×** **10**^**−5**^	2.0 (1.7)	0.23
Neuroticism	186	**−0.97 (0.19)**	**2.5** **×** **10**^**−7**^	−1.48 (0.77)	0.06	**−0.78 (0.22)**	**2.8** **×** **10**^**−4**^	−0.86 (0.70)	0.22
Leisure screen time	274	**−1.45 (0.11)**	**1.3** **×** **10**^**−37**^	−1.35 (0.44)	2.4 × 10^−3^	**−1.09 (0.13)**	**6.6** **×** **10**^**−17**^	−0.55 (0.44)	0.21
Educational attainment	544	**2.62 (0.12)**	**4.1** **×** **10**^**−99**^	**2.46 (0.41)**	**5.2** **×** **10**^**−9**^	**2.34 (0.16)**	**1.2** **×** **10**^**−45**^	1.66 (0.70)	0.018
Executive functioning	235	0.93 (0.34)	6.6 × 10^−3^	−0.62 (1.33)	0.64	1.09 (0.42)	9.8 × 10^−3^	2.00 (1.46)	0.17
Alzheimer	50	−0.1 (0.3)	0.79	−0.1 (0.5)	0.80	0.2 (0.4)	0.60	1.0 (0.8)	0.21
Drinks per week	124	−0.1 (0.31)	0.59	−1.0 (0.70)	0.16	−0.86 (0.35)	0.014	0.24 (0.87)	0.78
Cigarettes per day	78	**−0.62 (0.10)**	**9.9** **×** **10**^**−10**^	−0.07 (0.17)	0.68	−0.24 (0.13)	0.073	−1.01 (0.39)	0.012
Fruit	12	0.22 (0.88)	0.8	−4.05 (1.73)	0.042	−0.37 (0.95)	0.69	−1.42 (1.72)	0.43
Olive oil	12	0.08 (0.57)	0.89	−1.62 (1.44)	0.29	−0.35 (0.77)	0.65	−0.79 (1.35)	0.57
Vegetables	12	0.72 (1.10)	0.51	−1.16 (3.03)	0.71	1.71 (1.11)	0.12	2.00 (2.02)	0.34
Cheese	10	−1.22 (0.60)	0.042	−0.44 (1.50)	0.78	−1.53 (0.78)	0.050	−1.69 (1.24)	0.20
Inflammatory bowel disease	68	−1.08 (0.52)	0.035	−0.82 (2.79)	0.77	−0.95 (0.61)	0.12	−1.02 (1.20)	0.40
Cancer (lung)	18	−0.006 (0.044)	0.90	−0.06 (0.13)	0.66	0.016 (0.059)	0.79	0.066 (0.099)	0.52
Osteoarthritis	34	−4.5 (2.2)	0.036	−7.7 (5.8)	0.20	−0.5 (2.1)	0.81	3.3 (4.4)	0.46
Gastroesophageal reflux disease	110	**−0.94 (0.13)**	**1.2** **×** **10**^**−12**^	0.10 (0.49)	0.84	**−0.73 (0.13)**	**2.2** **×** **10**^**−8**^	−0.77 (0.43)	0.07

Significant results after multiple testing correction (*P* < 6.9 × 10^−4^) are highlighted in bold. Tests were two-sided.

#### MVMR analyses testing BMI as confounder

To understand the apparent protective effect of All-PA–leisure with respect to COVID-19 better, we considered a possible role of BMI as confounder, as high BMI leads to worse COVID-19 outcomes^20^. We performed a multivariable MR (MVMR) analysis, with the All-PA–leisure phenotype and BMI as exposures, and COVID-19 hospitalization as outcome. In this analysis, we still observed a significant protective effect of All-PA–leisure on the COVID-19 trait which considers hospitalized cases (effect = −0.067 ± 0.016, *P* = 2.8 × 10^−5^; Table 4). Considering the role that BMI has in many other health outcomes, we also conducted MVMR, including BMI for all other traits that had significant MR results. Despite the reduced power of the analysis due to a decrease in the number of instrumental variables suitable for all three traits, we found significant protective effects of All-PA–leisure on HDL-C, triglycerides, osteoarthritis and gastroesophageal reflux disease (Table 4 and Supplementary Table 26).

**Table 4:** MVMR analysis of All-PA–leisure setting BMI as exposures

Exposure	Outcome	*n*	*β* (s.e.)	*P* value
PA	Aneurysm (abdominal aortic)	19	−0.087 (0.055)	0.11
BMI	Aneurysm (abdominal aortic)	112	0.31 (0.18)	0.092
PA	COVID-19 hospitalization	**19**	**−0.067 (0.016)**	**2.8** **×** **10**^**−5**^
BMI	COVID-19 hospitalization	113	0.37 (0.05)	8.2 × 10^−13^
PA	asthma	19	−0.0069 (0.0026)	6.7 × 10^−3^
BMI	asthma	113	−0.0035 (0.0086)	0.69
PA	HDL-C	**19**	**0.0221 (0.0047)**	**2.3** **×** **10**^**−6**^
BMI	HDL-C	113	−0.042 (0.016)	7.0 × 10^−3^
PA	Triglycerides	**19**	**−0.038 (0.011)**	**3.6** **×** **10**^**−4**^
BMI	Triglycerides	113	0.040 (0.035)	0.26
PA	Type 2 diabetes	16	−0.058 (0.057)	0.30
BMI	Type 2 diabetes	101	0.42 (0.18)	0.19
PA	Parental survival	**18**	**0.030 (0.010)**	**2.9** **×** **10**^**−3**^
BMI	Parental survival	109	−0.171 (0.034)	4.4 × 10^−7^
PA	Phenotypic age acceleration	19	−0.091 (0.044)	3.7 × 10^−2^
BMI	Phenotypic age acceleration	113	0.92 (0.15)	4.8 × 10^−10^
PA	Osteoarthritis	**19**	**−0.0037 (0.0012)**	**1.4** **×** **10**^**−3**^
BMI	Osteoarthritis	113	0.0253 (0.0039)	1.3 × 10^−10^
PA	Gastroesophageal reflux disease	**19**	**−0.063 (0.015)**	**2.3** **×** **10**^**−5**^
BMI	Gastroesophageal reflux disease	113	0.141 (0.050)	4.6 × 10^−3^

For the instrumental variables, we used a threshold *P* = 10^−5^. Significant results after multiple testing correction (*P* < 5.0 × 10^−3^ outcomes) are highlighted in bold. Tests were two-sided.

#### All-PA–leisure and home and All-PA–leisure, home and work phenotypes

To evaluate the potential gain in information from combining data from PA during leisure time with PA performed at home and work, we conducted two additional GWAS in the EUR population. First, we summed All-PA–leisure with All-PA from PA at home (All-PA–leisure and home; Methods; Supplementary Fig. 11). We next added information from All-PA at work (All-PA–leisure, home and work; Methods; Supplementary Fig. 12). In both these GWASs, we observed a substantial reduction of the number of association peaks compared to All-PA–leisure.

### GWAS of vigorous PA phenotypes (leisure, at home and at work)

We ran analyses on different contexts of vigorous PA to understand genetic differences and benefits related to the context in which PA was performed.

#### Kinds of vigorous PA and their genetic interrelationships

We ran independent MVP GWAS of all vigorous activity types depending on the context (Vig-PA–leisure, Vig-PA–home and Vig-PA–work) in EUR and investigated correlations between each set of two to study their genetic differences. For Vig-PA–leisure and Vig-PA–home, *r*_*g*_ = 0.64 ± 0.04 (*P* = 5.0 × 10^−59^); Vig-PA–leisure and Vig-PA–work, *r*_*g*_ = 0.46 ± 0.07 (*P* = 9.8 × 10^−11^). Vig-PA–home and Vig-PA–work were more strongly correlated with each other (*r*_*g*_ = 0.97 ± 0.05, *P* = 1.2 × 10^−73^). SNP-*h*^2^ of vigorous PA phenotypes are reported in Supplementary Table 27.

For each vigorous PA trait, we ran GWAS, and for Vig-PA–leisure we also conducted X chromosome-wide association study (XWAS), gene-based testing, TWAS, fine-mapping, functional enrichment, mitochondria–SNV association and gene-based association analysis on the mitochondrial genome (Supplementary Results; Supplementary Tables 28–35 and Supplementary Figs. 13–17).

#### Genetic correlations of vigorous PA traits

**Fig. 3:**
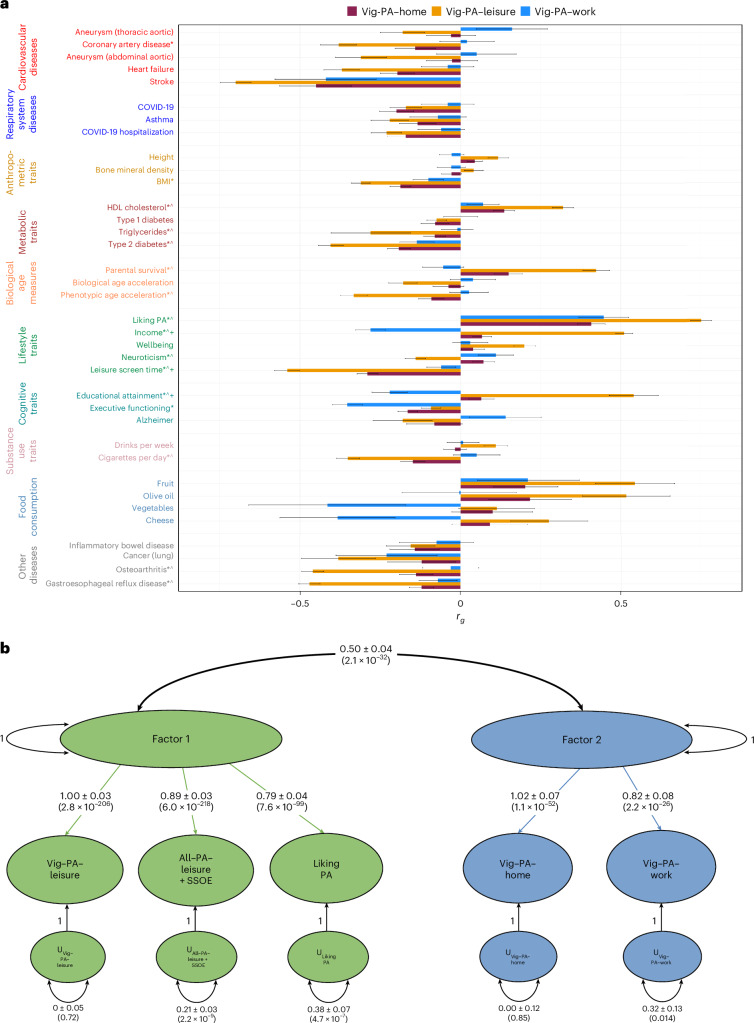
Genetic differences among vigorous PA types. **a**, Genetic correlation between each pair of vigorous PA contexts and other traits of interest. Significantly different correlations were defined as having *P* < 4.63 × 10^−4^ after multiple testing correction. An asterisk represents a significant difference between Vig-PA–leisure and Vig-PA–work, wedge represents a significant difference between Vig-PA–leisure and Vig-PA–home, and plus represents a significant difference between Vig-PA–home and Vig-PA–work. Tests were two-sided. Black error bars represent the s.e. The genetic correlation values of the vigorous PA with the other traits of interest are provided in Supplementary Tables 36–38. **b**, Genomic-SEM of the two-factor model, showing the loadings for the inferred traits—their standardized estimates with the s.e. in parentheses. The CFA uses a chi-square test.

To understand the differences between the vigorous activity traits better, we calculated the genetic correlations between Vig-PA–leisure, Vig-PA–work and Vig-PA–home individually with the previously analyzed traits of interest (Supplementary Tables 36–38). We observed more statistically significant differences between Vig-PA–leisure and Vig-PA–work (for 15 traits), than when considering Vig-PA–leisure and Vig-PA–home (for 12 traits) or Vig-PA–home and Vig-PA–work (for 3 traits; Fig. 3a). Among these three pairs of vigorous activity traits, there were numerous significant differences regarding genetic correlations with income, leisure screen time and educational attainment, reporting better health benefits from Vig-PA–leisure compared to the other two vigorous traits. Analyses performed adjusting the vigorous PA traits with income using mtCOJO are described in Supplementary Results, Supplementary Table 39 and Supplementary Fig. 18.

#### Genomic structural equation modeling (genomic-SEM) analysis

Considering the differences identified among the three vigorous PA traits, which had shown different benefits according to the context, we used genomic-SEM analysis^21^ to understand their relationship better, inferring the overall genetic architecture among them and two other PA traits in EUR, All-PA–leisure + SSOE and liking of PA. The exploratory factor analysis (EFA) indicated that a two-factor model fit the data best (Fig. 3b), accounting for 91% of the cumulative variance explained (factor 1, 50%; factor 2, 41%). Both factors had strong strength of sum of squares (SS) loadings (factor 1 SS = 2.50; factor 2 SS = 2.04). Confirmatory factor analysis (CFA) showed that two correlated factors model fit the data well, with comparative fit index = 0.97, chi-square = 109.03, Akaike information criterion = 129.03 and standardized root mean square residual = 0.069. Vig-PA–leisure (loading = 1.00 ± 0.03), All-PA–leisure + SSOE (loading = 0.89 ± 0.03) and liking of PA (loading = 0.79 ± 0.04) loaded most strongly on factor 1 (leisure/liking PA), while Vig-PA–home (loading = 1.02 ± 0.07) and Vig-PA–work (loading = 0.82 ± 0.08) loaded most strongly on factor 2 (occupational/domestic PA; Fig. 3b). The factor leisure/liking PA and factor occupational/domestic PA were correlated at 0.50 (0.04).

#### Survival analyses

Survival analyses showed a significant reduction of risk of all-cause death for Vig-PA–leisure (*β* = −0.1173 ± 0.0037; *P* ≤ 2 × 10^−16^) and for Vig-PA–work (*β* = −0.0061 ± 0.0029; *P* = 0.036). There was no statistically significant reduction of risk of death for Vig-PA–home, though the direction of effect and effect size were similar (*β* = −0.0052 ± 0.0030; *P* = 0.082).

## Discussion

PA has long been appreciated to ameliorate the risk of illness. Increasing PA on a population level could greatly reduce morbidity and mortality—this is a major public health undertaking and very difficult to accomplish. Understanding genetic factors that influence the level of activity for individuals could provide pathways for interventions to support efforts to increase PA. We present here comprehensive information on the genetics of PA and investigate the relationships between PA and health. This work provides tools to dissect causal relationships between a range of other traits and PA, and to uncover previously unknown biology responsible in part for interindividual differences in PA. Those relationships were remarkably different depending on the context of the PA—in particular, whether the activity was associated with PA during leisure time (protective) rather than at work or around the house (often not protective). Our main focus was accordingly on leisure-PA. All-PA–leisure considered different levels of PA intensity and frequency. We completed a GWAS of All-PA–leisure for EUR, AFR and AMR ancestry in MVP. We combined these data with the UKB SSOE trait in two meta-analyses, one with EUR individuals and the other cross-ancestry.

The polygenic architecture of leisure-time PA showed that genetic liability to PA was protective with respect to numerous diseases. It was previously reported that PA has negative genetic correlation with type 2 diabetes^13,15,22^, triglyceride level^13^, rheumatoid arthritis^15^, coronary artery disease^15^ and lung cancer^15^. There is prior genetic evidence of the role of PA in preventing colorectal cancer using MR methods^23^, but we did not find a significant genetic correlation with colorectal cancer. Our analyses confirmed the negative genetic correlations with type 2 diabetes, coronary artery disease and lung cancer. Moreover, we found significant negative genetic correlations between PA and other medically relevant traits—thoracic and abdominal aortic aneurysm, heart failure, stroke, asthma, type 1 diabetes, Alzheimer’s disease, inflammatory bowel disease, osteoarthritis and gastroesophageal reflux disease. PA was also protective with respect to COVID-19 and COVID-19-associated hospitalization, and positively correlated with parental survival.

Whereas genetic correlations are an average of the shared association across the genome, local genetic correlations can detect regions of shared genetic associations, including finding signals in opposing directions that a genome-wide analysis would not capture. Local genetic correlation, like global correlation, supported a protective role of PA for multiple health traits, showing negative local genetic correlations for diabetes types 1 and 2, gastroesophageal reflux disease and osteoarthritis. We also found negative local genetic correlations between All-PA–leisure + SSOE and BMI, triglycerides, phenotypic age acceleration, leisure screen time, neuroticism and cigarettes per day. Positive local genetic correlations were found between All-PA–leisure + SSOE and bone mineral density, income, liking of PA, educational attainment, and, unexpectedly, oral cancer—there was a single locus showing positive local genetic correlation between PA and oral cancer; however, genome-wide genetic correlation between these traits calculated with LDSC was not significant (*P* = 0.418). We also found local genetic correlations in opposite directions—at different genomic locations—with All-PA–leisure + SSOE for height and well being.

Our MR results confirmed a protective effect of All-PA–leisure genetic liability against type 2 diabetes, abdominal aortic aneurysm, heart failure, COVID-19-associated hospitalization, triglyceride level, osteoarthritis and gastroesophageal reflux disease. Most of these causal effects were observed even when we tested for BMI as a confounder in the MR (MVMR; except for heart failure, which could not be tested due to sample overlap between the BMI and heart failure datasets). However, the low number of instrumental variables in our MVMR analyses could result in false negatives. We tested BMI as confounder since it is genetically correlated to numerous diseases and a previous study showed that it is likely a mediator of leisure time PA, as its causal effect on leisure time PA disappears when taking education into account^24^. These findings nevertheless confirm the utility of All-PA–leisure to decrease risk for numerous diseases^25,26^, independent of the role of BMI as either a mediator or a confounder. MR results also suggested a protective effect of All-PA–leisure on the biological age measure phenotypic age acceleration, and a bidirectional effect between All-PA–leisure and parental survival, a protective effect of genetic liability for this PA phenotype versus aging and mortality.

PheWAS analysis in BioVU^27^, a broad exploration of disease traits in an independent sample, showed a negative correlation of PA with several previously mentioned health traits, including diabetes, cardiovascular disease (for example, coronary artery disease and heart failure), gastroesophageal reflux disease, lung cancer and asthma. The strongest association was related to diabetes (*P* = 5.89 × 10^−^^36^).

The EUR meta-analysis of All-PA–leisure + SSOE identified 67 lead SNPs, 59 of which were novel compared to the lead SNPs of the two GWASs, All-PA–leisure and SSOE, in the meta-analysis. Previous GWAS of self-report and device-measured PA identified 68 lead variants in total^13–15^. We replicated associations for 17 previously identified lead variants. Two (CADM2*rs62253088 and LOC642366*rs4865656) were still lead variants in both our meta-analyses of All-PA–leisure + SSOE (EUR ancestry and cross-ancestry); the other 15 were significantly associated (Supplementary Table 40). CADM2*rs62253088 (encoding *Cell Adhesion Molecule 2*), on chromosome 3, had the strongest association in both EUR and cross-ancestry All-PA–leisure + SSOE meta-analyses (EUR meta-analysis *P* = 2.0 × 10^−^^21^; cross-ancestry (EUR, AFR and AMR) meta-analysis *P* = 6.1 × 10^−^^20^), but was not significant in the GWAS of Vig-PA–leisure. However, considering Vig-PA–leisure, another variant mapping to *CADM2* was a significant lead SNP in the GWAS (rs2326266; *P* = 4.1 × 10^−^^8^) and *CADM2* also had the most significant *P* value in the gene-based test (2.5 × 10^−^^8^). *CADM2* variants, including rs62253088, have previously been associated with PA, and also with decreased neuroticism and decreased self-reported nervous and anxious feelings^13^. *CADM2* was also previously found to be associated with obesity and metabolic traits^28^, cannabis use^29^, smoking and alcohol related traits^30^, risk-taking behavior^31^, educational attainment^32^ and autism spectrum disorder^33^. *CADM2* encodes a member of the synaptic CADM (SynCAM) family, which belongs to the immunoglobulin superfamily^34^. Through a previous summary-based MR^35^ analysis of eQTL signals, *CADM2* was suggested to influence PA through effects on skeletal muscle^15^.

Significant variants also included MST1R*rs3733134 (EUR meta-analysis *P* = 1.4 × 10^−^^17^; cross-ancestry meta-analysis *P* = 6.9 × 10^−^^18^), EXD2*rs4899292 (EUR meta-analysis *P* = 1.7 × 10^−16^; cross-ancestry meta-analysis *P* = 3.8 × 10^−^^16^), *LONRF2* and *SLC39A8*. *SLC39A8* was previously associated with several psychiatric disorders, including problematic alcohol use^36^, schizophrenia^37^ and opioid use disorder^38^ (Supplementary Discussion).

Additional analyses demonstrated that PA is related to brain structure and cellular architecture. From MAGMA tissue expression analyses, the cerebellum and cerebellar hemisphere showed significant enrichments. Cerebellar function is critical for skills relevant to PA, such as coordination and movement. These results were similar to the results of the previous GWAS of SSOE^13^. Cell-type-specificity analyses showed a significant association of PA with GABA^+^ neurons^39^. EUR All-PA–leisure + SSOE and cross-ancestry meta-analyses shared the following three significant gene sets in their MAGMA gene-set analysis results: structural constituent of presynapse, presynaptic active zone organization, and maintenance of presynaptic active zone structure. This finding highlights the role of presynaptic cells in PA and the importance of the cerebellum and GABAergic function in the biology of PA levels.

Vig-PA–leisure was, as noted, genetically different from Vig-PA–home and Vig-PA–work. We observed moderate genetic correlation between Vig-PA–leisure and Vig-PA–home (*r*_*g*_ = 0.64 ± 0.04, *P* = 5.0 × 10^−^^59^), but lower genetic correlation between Vig-PA–leisure and Vig-PA–work (*r*_*g*_ = 0.46 ± 0.07, *P* = 9.8 × 10^−^^11^). While there was high genetic correlation between Vig-PA–home and Vig-PA–work (*r*_*g*_ = 0.97 ± 0.05, *P* = 1.2 × 10^−^^73^), there were differences between these two traits in genetic correlation analyses with other traits of interest, in particular for income, educational attainment and leisure screen time. Previous work on dementia also showed genetic differences between PA during leisure versus work time^40^.

The genomic-SEM results also supported that context-defined PA traits are genetically heterogeneous. Traits more similar to voluntary PA and healthy lifestyle habits loaded on the leisure/liking PA Factor, while the occupational/domestic PA Factor captured traits more consistent with strenuous work and labor performed as part of work duties and responsibilities at home.

While the leisure/liking PA and occupational/domestic PA factors were moderately correlated (0.50), the nature of the respective contexts of PA identified key differences. Perhaps the most notable distinction is that strenuous occupational and domestic PA is often not health-enhancing, and can be detrimental to health via injury and long-term stress on bodily systems^41,42^. Individuals whose vocations are labor-intensive are also less likely to engage in health-promoting PA in leisure^43^, further compounding their risk for adverse health outcomes long-term.

When we adjusted for vigorous PA traits and income using mtCOJO, we still observed differences in genetic correlations with health traits, suggesting that these differences do not merely reflect differences in socioeconomic status. These findings together suggest that not all contexts of PA are equally beneficial, and some forms of vigorous activity may be more strongly linked than others to improved mental and physical health.

A further confirmation of differing benefits by PA context was provided by the survival analyses, which show a strong significant reduction of risk of death for Vig-PA–leisure (*β* = −0.1173 ± 0.0037, *P* ≤ 2 × 10^−^^16^) compared to a weak effect for Vig-PA–work (*β* = −0.0061 ± 0.0029, *P* = 0.036), and none for for Vig-PA–home (*β* = −0.0052 ± 0.0030, *P* = 0.082). The small protective effect for Vig-PA–work contrasts with the opposing directions for risk for several other important health traits. The survival analyses may be confounded by health status (healthier individuals are able to exercise more vigorously) and competing risks (death). A previous study on different kinds of PA during leisure time—for example, weightlifting and aerobic exercise—showed, consistent with the present report, that PA in the leisure context can lead to a longer and healthier life^44^. We conclude that PA at leisure is the most protective with respect to deleterious medical outcomes.

This study has limitations, including a relative lack of females in the MVP dataset (approximately 9%, which nevertheless equates to >17,000 females for Vig-PA–leisure); this makes the interpretation of sex-specific associations infeasible. UKB, though, has excellent female representation. MVP is also exclusively based on military veterans, not fully representative of the general population. However, the high genetic correlation (*r*_*g*_ = 0.76 ± 0.03, *P* = 4.7 × 10^−^^118^) between MVP data (All-PA–leisure) and SSOE in UKB, a more representative cohort, may also be considered in this context. Moreover, while there are many non-EUR participants included, their numbers are still insufficient, so the cross-ancestry analysis mostly reflects findings in EUR. It would also be important to verify the tissue expression analyses, which highlight only significant associations with the brain, with more tissues and larger samples. Our results may reflect a lack of power for other tissues or our sample size—of the 53 tissue types contained in Genotype-Tissue Expression version 8 (GTEx v8), 13 are brain-specific.

In conclusion, we have made progress towards revealing the genetic and biological nature of PA, which can differ greatly depending on the context. In effect, the PA that one chooses to engage in in a leisure context has many positive effects with respect to health traits, while activity that one must do, in a work or household context, tends not to have these beneficial effects. This difference persisted even after we adjusted for income. Moreover, PA in the leisure context shows a much stronger decrease in the risk of death compared to the other contexts. This may reflect the differences in intensity and duration of leisure activities compared to work and home activities. We detected a protective role of PA genetic liability on several diseases, using global and local genetic correlation, PheWAS analyses and causal inference using MR. MR analyses showed significant protective effects of PA on several traits, including type 2 diabetes, abdominal aortic aneurysm, heart failure, triglyceride level, osteoarthritis, gastroesophageal reflux disease and COVID-19-associated hospitalization. These results provide genetic insights into the biological bases of PA and add to strong experimental, epidemiological and observational literature showing the value to health of PA.

## Methods

### MVP datasets

The U.S. Department of Veterans Affairs (VA) MVP is one of the largest and most diverse biobanks in the world, with genetic and electronic health record (EHR) data available^45,46^. Ethical approval for the MVP study was obtained from the Central VA Institutional Review Board and the site-specific institutional review boards. All relevant ethical regulations for work with human participants were followed in the conduct of the study, and informed consent was obtained from all participants.

Participants provided a blood sample for genomic analyses, granted access to medical records, and a subset agreed to complete two questionnaires, the MVP Baseline and Lifestyle Surveys. PA information used in this study was collected from the MVP Lifestyle Survey, and data were subdivided according to context (leisure, work and home), intensity (vigorous, moderate and light) and frequency (Supplementary Fig. 1). The three levels of activity were defined as follows:

1. Vigorous—activities that cause your heart to beat rapidly and you work up a good sweat, and are breathing heavily; performed at least 10 min at a time.
2. Moderate—activities that cause your heart rate to increase slightly and you typically work up a sweat, but are not physically exhausting; performed for at least 10 min at a time.
3. Light—activities that require little physical effort.

The levels of activity were considered together with frequency information, which could be ‘daily’, ‘several times per week’, ‘once per week’, ‘several times per month’, ‘once per month or less’ or ‘never’, when participants answered three questions regarding the place where PA was effected (Supplementary Methods). The three different levels of PA were summed considering All-PA phenotype frequency and weighting intensity to create a total score, reported in the Supplementary Methods.

From previous MVP analyses, after MVP genotyping performed using a customized Affymetrix Axiom Biobank Array and quality control^47^, EAGLE2 (ref. ^48^) was used for phasing chromosomes and Minimac3 was used for imputation^49^, using the 1000 Genomes Project reference panel, Phase 3, version 5 (ref. ^50^). Populations were defined using principal component analysis^45^.

For All-PA during leisure time (All-PA–leisure), we ran independent GWAS for each of the three ancestries (EUR, AFR and AMR; Supplementary Fig. 1). For EUR, we also ran GWAS for vigorous PA during leisure time (Vig-PA–leisure), work time (Vig-PA–work) and home time (Vig-PA–home). We did not have enough power to run GWAS for vigorous PA for AFR and AMR ancestries.

In the quality control procedure for GWAS conducted using PLINK 2.0 (ref. ^51^), we removed variants with imputation quality scores < 0.6, Hardy–Weinberg equilibrium *P* < 5 × 10^−5^, minor allele frequency <0.01, missing call rates for variants >0.1 and missing call rates for samples >0.1. Data were aligned to the GRCh37 reference genome. Considering EUR ancestry, after filtering, we had 6,660,206 variants for All-PA–leisure phenotype, and 6,548,052, 6,536,056, 6,537,158 variants for Vig-PA–leisure, Vig-PA–home and Vig-PA–work, respectively. For AFR and AMR ancestries, we kept 11,984,943 and 7,969,366 variants, respectively (All-PA–leisure phenotype).

To remove related individuals, we used a threshold of 0.0884 for the kinship coefficients calculated by KING^52^, resulting in the removal of individuals with a minimum of a second-degree relationship. Then, we implemented an algorithm to optimize retaining the more informative individuals, keeping the maximum number of individuals with the highest score (for All-PA–leisure phenotype) or with the highest frequency (for the vigorous activity phenotypes). If two individuals had the same score (or frequency), we removed the one with the highest number of relationships. For EUR, the final sample sizes were as follows: 189,812 individuals for the All-PA–leisure phenotype, 201,050 for Vig-PA–leisure, 203,430 for Vig-PA–home and 171,278 for Vig-PA–work. For AFR and AMR ancestries, we kept 27,044 and 10,263 individuals, respectively (All-PA–leisure phenotype). We ran GWAS analysis using a linear regression model implemented in PLINK 2.0, using sex, age and the first ten principal components as covariates.

For All-PA–leisure&home phenotype and for All-PA–leisure&home&work, we ran the same QC as described in the Methods, using EUR ancestry. After filtering, we had 6,678,873 variants for All-PA–leisure&home, and 6,761,070 variants for All-PA–leisure&home&work. For the final sample size, where we excluded individuals without full information on their PA, we retained 181,317 individuals for All-PA–leisure&home, and 146,929 for All-PA–leisure&home&work.

### UKB summary statistics

UKB is a cohort study of ~500,000 adults aged 40–69 years in the United Kingdom (UK) and was recruited from 22 centers across the country^53^. All UKB participants provided written informed consent^53^. Ethical approval of the UKB study was given by the North West Multicentre Research Ethics Committee, the National Information Governance Board for Health and Social Care and the Community Health Index Advisory Group^53^.

From the UKB public databases, we downloaded GWAS summary statistics for the SSOE phenotype with the accession code GCST006100, for MVPA with the accession code GCST006097 and for VPA with the accession code GCST006098 (ref. ^13^). Self-reported information was reported via a touchscreen questionnaire. For SSOE, participants needed to answer the following question: ‘in the last 4 weeks, did you spend any time doing the following?’. Then, there were follow-up questions assessing the frequency and typical duration of ‘strenuous sports’ and of ‘other exercises’. Cases were defined as individuals spending 2–3 days per week or more doing strenuous sports or other exercises for a duration of 15–30 min or greater, and controls as those individuals who did not indicate spending any time in the last 4 weeks doing either SSOE, obtaining 124,842 cases (*n*_case_) and 225,650 controls (*n*_control_)^13^. We defined the population effective size as *n*_eff_ = 4/((1/*n*_case_) + (1/*n*_control_)).

The downloaded summary statistics had SNPs filtered according to—Hardy–Weinberg equilibrium *P* < 10^−6^, high missingness >1.5%, low minor allele frequency <0.1%^13^. We also filtered low imputation quality <0.6, yielding approximately 11.7 million available SNPs.

### Meta-analyses

METAL^54^ was used for EUR ancestry meta-analysis, which included EUR MVP data for the All-PA–leisure phenotype and UKB summary statistics for the SSOE phenotype^13^, in an All-PA–leisure + SSOE meta-analysis. We also ran a cross-ancestry All-PA–leisure + SSOE meta-analysis, adding AFR and AMR summary statistics from MVP data to the EUR All-PA–leisure + SSOE meta-analysis (Supplementary Fig. 1). We conducted the meta-analyses using the sample-size method, which considers *P* value and direction of effect, weighted according to sample size. We used this method to combine summary statistics from a logistic regression model (UKB summary statistics for SSOE phenotype) and a linear regression model (MVP summary statistics for All-PA–leisure phenotype).

### XWAS

Sex-stratified analysis of the X chromosome was conducted in the EUR MVP sample using XWAS 3.0 on hard-call genotypes^55^. We included 174,375 males and 15,130 females for the All-PA–leisure phenotype and 229,331 males and 17,072 females for Vig-PA–leisure. Following sex-stratified analysis, test statistics from males and females were combined using Stouffer’s method^56^. Variants with Hardy–Weinberg equilibrium *P* < 5 × 10^−^^6^ were filtered; 191,114 variants were included for the All-PA–leisure phenotype and 191,148 for Vig-PA–leisure. We used age and the first ten principal components as covariates.

### Gene-based and gene-set analyses, functional enrichment

Using the tool MAGMA^57^, implemented in the FUMA web-based platform^58^, we annotated variants and ran gene-based tests, gene-set analyses and functional enrichment for each of the two meta-analyses (EUR ancestry and cross-ancestry) for the All-PA–leisure phenotype and for the GWAS of Vig-PA–leisure. For annotation, we used 1000 Genome Phase 3 EUR for EUR ancestry analyses and 1000 Genome Phase 3 ALL for the cross-ancestry All-PA–leisure+SSOE meta-analysis. The other parameters for the identification of lead SNPs were a maximum cutoff *P* < 0.05, *r*^2^ threshold to define independent significant SNPs >0.6, a second *r*^2^ threshold to define lead SNPs >0.1 and a maximum distance between linkage disequilibrium (LD) blocks to merge into a locus <250 kb.

The MAGMA gene-based test is based on a multiple regression approach to detect gene effects, considering SNP *P* values and linkage disequilibrium. We also ran a gene-based approach on our EUR All-PA–leisure + SSOE meta-analysis using fastENLOC^17,18^ (fast enrichment estimation aided colocalization analysis). This method was used to prioritize likely causal gene–trait associations with precomputed eQTL annotations from the 49 tissues in the GTEx v8 dataset. We kept as ‘noteworthy genes’ those with GLCP ≥ 0.9; if a gene was colocalizing among two or more tissues, we retained the tissue with the highest GLCP value.

Gene-set analyses are performed for curated gene sets and GO terms obtained from MsigDB, with terms having a Bonferroni-corrected *P* < 0.05 considered significant^57^. Gene property analyses were performed for tissue-specific gene expression using GTEx v8 dataset^59^.

### Single-cell expression

FUMA also includes a tool to test cell-type-specificity analysis^60^, which was run using the following datasets of human samples: Allen Brain Atlas Cell Type^61^, DroNc^62^, GSE104276 (ref. ^63^), GSE67835 without fetal samples^64^, GSE81547 (ref. ^65^), GSE84133 (ref. ^66^), GSE89232 (ref. ^67^), GSE101601 (Linnarsson’s lab)^68^, GSE76381 (Linnarsson’s lab)^69^ and PsychENCODE^70^.

### SNP-*h*^2^ and genetic correlation

LDSC^16^ was used to calculate SNP-*h*^2^ for EUR ancestry and to calculate genetic correlation^71^ between the EUR meta-analysis of the All-PA–leisure phenotype and other traits, to quantify genetic similarity (Supplementary Methods ‘Traits studied for genetic correlation with PA’). To determine which traits were significant, we used the Benjamini–Hochberg procedure (Supplementary Table 15; ‘Benjamini–Hochberg false discovery procedure’). To estimate SNP-*h*^2^ for AFR and AMR cohorts, we calculated LD scores with cov-LDSC^72^ from 10,000 random independent individuals from MVP filtering the SNPs to keep only those that were previously identified in the HapMap Project^73^. We conducted multitrait conditional and joint analysis to evaluate the potential confounding of socioeconomic status with mtCOJO (Supplementary Notes).

### Benjamini–Hochberg false discovery procedure

To determine which traits were significantly genetically correlated with PA, we used the Benjamini–Hochberg procedure (Supplementary Table 19)^74^. We ranked the traits according to their increasing *P* value, then we calculated the *P* value’s Benjamini–Hochberg critical value by multiplying each rank by 0.05 and dividing it by the total number of analyzed traits. Finally, the trait *P* values were compared to the critical values, considering a trait significant until the largest *P* value was smaller than its corresponding critical value.

### Local genetic correlation

We ran LAVA^19^ to calculate local genetic correlations between the EUR All-PA–leisure + SSOE meta-analysis and the 41 traits (Supplementary Table 41) also studied for genome-wide (global) genetic correlation, to identify shared genetic bases in specific genomic regions. LAVA allows estimation of local genetic heritability and local genetic correlations. We considered 2,495 genomic loci, defined by partitioning the genome into blocks of around 1 Mb and minimizing the LD between the blocks^19^. Based on the number of genomic loci, we defined significant local genetic heritability as having a Bonferroni-corrected *P* < 0.05/2,495. We evaluated local genetic correlation at loci with pairs of traits that have already been shown to have significant local genetic heritability for both phenotypes. Thus, we conducted 3,088 local genetic correlation tests, defining significance as those with a Bonferroni-corrected *P* < 0.05/3,088.

### MR

MR analyses enable the inference of causality between traits with genetic similarity. Two-sample MR was performed using the following four methods: MR-Egger, weighted median, IVW and simple mode^75^. For the PA trait, we used EUR ancestry MVP data of the All-PA–leisure phenotype to account for problems of population stratification and of overlapping samples. We filtered SNPs with *P* > 10^−5^ and clumping data using the default window of 10,000 kb and the *r*^2^ cutoff of 0.001. To designate significant results, we applied multiple testing correction for 36 outcomes used both as outcomes and as exposures (*P* = 6.9 × 10^−4^). We also used TwoSampleMR to run MVMR analyses of the All-PA–leisure phenotype as exposure, to understand the influence that BMI could have as a confounder, setting it as exposure (Supplementary Notes).

### TWAS

TWAS was used to identify genes associated to traits. We ran TWAS using FUSION^76^ with the 1000 Genomes LD reference data (https://data.broadinstitute.org/alkesgroup/FUSION/LDREF.tar.bz2) and the GTEx v8 multitissue expression weights from 49 tissues (https://www.mancusolab.com). These tissues included adipose, adrenal gland, artery, brain, breast, skin, blood, colon, esophagus, heart, kidney, liver, lung, minor salivary gland, muscle, nerve, ovary, pancreas, pituitary, prostate, small intestine, spleen, stomach, testis, thyroid, uterus and vagina. We performed expression imputation for each autosome using these tissue weights; thus, we identified genes that were conditionally independent. Based on these 49 tissue weights and a total of 27,977 genes, we used a multiple testing correction of 3.65 × 10^−^^8^. If a gene was significant and expressed in at least two tissues, we chose the gene expression with the lowest *P* value.

### Fine-mapping

Fine-mapping of causal gene sets was used to fine-map TWAS at genomic risk regions, identifying likely causal genes^77^. We used the All-PA–leisure + SSOE EUR meta-analysis and the Vig-PA–leisure as input GWAS, and the GTEx v7 weights from PrediXcan^78^ combined with Metabolic Syndrome in Men Study^79^, Netherlands Twins Registry^80^, Young Finns Study^81^ and CommonMind Consortium^82^ weights. LD scores were obtained from the 1000 Genomes Phase 3.

We also conducted fine-mapping analyses at the SNP level using Polygenic functionally informed fine-mapping^83^, using precomputed prior causal probabilities based on a meta-analysis of 15 UKB traits, allowing for the extraction of per-SNP heritabilities (SNPVAR). These were necessary together with the summary statistics and EUR genotypes from 1000 Genomes Project Phase 3 to perform functionally informed fine-mapping with the sum of single effects^84,85^.

### Multitrait analysis of GWAS (MTAG)

This method was employed to facilitate joint analysis of summary statistics from GWAS of different but related traits, such as the MTAG^86^, to increase power in GWAS analyses. Here we ran two MTAG analyses, both with the EUR All-PA–leisure + SSOE meta-analysis as a single trait. First, we joined this trait with leisure screen time^15^. Because of their negative genetic correlation, we flipped the allele in the LSC statistics. Second, we joined the EUR All-PA–leisure + SSOE meta-analysis with a measure of PA-liking^87^.

### Enrichment analysis

g:Profiler is a toolset that also includes finding biological categories enriched in a gene list^88^. We performed it on three gene lists resulting from the FUMA gene-based test—EUR All-PA–leisure+SSOE meta-analysis, cross-ancestry All-PA–leisure+SSOE meta-analysis and Vig-PA–leisure.

### Mitochondrial genome association analyses

We used mitochondria genomes of EUR ancestry individuals from MVP to run mitochondria–SNV association analyses and gene-based association analyses with All-PA–leisure and Vig-PA–leisure phenotypes. For the mitochondria–SNV association analyses, we filtered missing call rates for variants >0.1, missing call rates for samples >0.1, and we excluded monomorphic variants. We thus analyzed 141 SNVs from 189,782 individuals for All-PA–leisure and 142 SNVs from 201,018 individuals for Vig-PA–leisure. For the gene-based association analyses, we initially assigned the SNVs to mitochondrial genes using the reference sequence NC_012920.1 (ref. ^89^); each SNV mapped to a single gene, and then we used the R software SKAT v2.2.4 to perform associations with All-PA–leisure and Vig-PA–leisure phenotypes, using age, sex and the first ten principal components as covariates^90^. We considered the nominally significant *P* value (≤0.05).

### PheWAS

We used Vanderbilt University Medical Center’s (VUMC) EHR database to conduct a PheWAS as an empirical exploration of PA and its related phenotypes, investigating a broader range of traits compared to those used in the genetic correlation analyses. The VUMC EHR database, also known as the Synthetic Derivative, houses de-identified, longitudinal medical histories of over 3.1 million patients, including their demographic information (age, sex and ethnicity), and clinical information like medications, lab values, procedural and surgical codes and diagnostic codes^27^. Codes from both the International Classification of Diseases, ninth and tenth editions (ICD-9 and ICD-10), are used by healthcare professionals to code symptoms, procedures and diagnoses in the synthetic derivative. A subset of the patients from the Synthetic Derivative also has their biological data associated with their medical records. This subset is referred to as BioVU and was used for genomic and phenomic analyses^91–93^.

For our analysis, we performed a PheWAS across BioVU records using a polygenic score for PA derived from the EUR All-PA–leisure + SSOE meta-analysis. First, we used PRS-CS^94^ to calculate a polygenic score for PA for 66,917 BioVU records of EUR participants with genotyped data. Next, using the PA polygenic score as the continuous predictor, we ran a PheWAS^95^ to explore the phenomic landscape of PA. The first step in PheWAS involves mapping related ICD codes to their phecode using the R PheWAS package^96^. The complete list of phecodes, along with the corresponding ICD-9 and ICD-10 codes mapped to them, can be found on the PheWAS catalog (https://phewascatalog.org/). Finally, using the EHR-driven case-control status for each phecode, we estimated the association between the PA polygenic score and the phecodes. We restricted our analysis to 1,254 phecodes that meet the following three criteria: (1) phecodes need over 100 cases or control, (2) phecodes should not be specific to a single sex and (3) phecodes should not be included in the broader mental health phenotypes. We also controlled for the first ten ancestry principal components, median age of the record and reported sex.

### Genomic structural equation modeling

Genomic structural equation modeling (genomic-SEM) was used to evaluate the overall genetic architecture of the PA traits included in the EUR GWAS analyses^21^. Summary statistics for the All-PA–leisure + SSOE meta-analysis, and for GWAS of Vig-PA–leisure, Vig-PA–home, Vig-PA–work and liking of PA were used to perform EFA and CFA. EFA model fit was evaluated by the amount of cumulative variance explained, the strength of SS loadings (≥1), and balance in the proportion of variance explained by each of the individual factors. Traits with factor loadings ≥0.20 in the EFA were allowed to load on the respective factors and evaluated for CFA model fit as determined by conventional fit indices^21^.

### Survival analyses

We ran the Cox proportional-hazards model using the package survival 3.3-1 in *R*, to establish the impact of vigorous PA for the three contexts (leisure, home and work) on the risk of dying of any cause. Information was collected in the MVP dataset for age, censoring status, and vigorous PA. Age was calculated for censored individuals from the current year minus the year of birth; for dead participants, the year of death was provided. The vigorous PA phenotypes present in MVP were previously described in the Methods ‘MVP datasets’ as follows: we considered Vig-PA–leisure, Vig-PA–home and Vig-PA–work classification. We had complete information for 252,718 individuals (of whom 55,849 were deceased) for Vig-PA–leisure, 253,096 individuals (of whom 55,203 were deceased) for Vig-PA–home and 207,862 individuals (of whom 43,294 were deceased) for Vig-PA–work.

### Reporting summary

Further information on research design is available in the Nature Portfolio Reporting Summary linked to this article.

## Online content

Any methods, additional references, Nature Portfolio reporting summaries, source data, extended data, supplementary information, acknowledgements, peer review information; details of author contributions and competing interests; and statements of data and code availability are available at 10.1038/s41588-025-02260-9.

## Supplementary information

Supplementary Information Supplementary Notes (Supplementary Methods, Supplementary Results and Supplementary Discussion), Supplementary Figs. 1–20 and VA Million Veteran Program core acknowledgement.

Reporting Summary

Supplementary Tables 1–41 Supplementary Tables 1–41.

## Data Availability

All MVP and meta-analysis summary statistics are made available through CIPHER https://phenomics.va.ornl.gov/web/cipher/partner/mvp: email CIPHER@va.gov with title ‘MVP and meta-analyses Summary Results–Galimberti et al.’.

## Code availability

Codes for software and packages used in this analysis are all publicly available through the citations for each method as introduced.
