# Supplementary material for: A contextual genomic perspective on physical activity and its relationship to health, well being and illness
Source: Nat Genet. 2025 Jul 21;57(8):1860–71. doi: 10.1038/s41588-025-02260-9 (PMC12339386; doi:10.1038/s41588-025-02260-9)
Supplement: Supplementary file 1 — Supplementary Notes (Supplementary Methods, Supplementary Results and Supplementary Discussion), Supplementary Figs. 1–20 and VA Million Veteran Program core acknowledgement. [file 41588_2025_2260_MOESM1_ESM.pdf]

nature genetics

Supplementary information https://doi.org/10.1038/s41588-025-02260-9

A contextual genomic perspective on physical activity and its relationship to health, well being and illness

In the format provided by the authors and unedited

Supplementary Information

Supplementary Notes

- Supplementary Methods
  1. MVP datasets
  2. MVP All-PA-Leisure GWAS validation
  3. MVP All-PA-Leisure score transformation
  4. Traits studied for genetic correlation with PA

- Supplementary Results
  1. MVP All-PA-Leisure GWAS validation
  2. SNP heritability and genetic correlation of PA phenotypes
  3. TWAS and fine-mapping analyses of All-PA-Leisure+SSOE meta-analyses
  4. Gene-based association analyses, gene-set analyses, and functional enrichment of cross-ancestry All-PA-Leisure+SSOE meta-analysis
  5. Mitochondrial genome association analysis of EUR All-PA-Leisure
  6. Genetic correlation of EUR All-PA-Leisure+SSOE with traits of interest
  7. PheWAS of All-PA-Leisure+SSOE
  8. Mendelian Randomization analyses of All-PA-Leisure
  9. Genes detected by gene-based approaches
  10. MTAG analyses of EUR All-PA-Leisure+SSOE
  11. Kinds of vigorous PA and their genetic interrelationships
  12. Multi-trait conditional and joint analysis of vigorous PA traits

- Supplementary Discussion

Supplementary Figures

- *Supplementary Figure 1:* Overview of the study design
- *Supplementary Figure 2:* GWAS analysis of the All-PA-Leisure phenotype in MVP EUR
- *Supplementary Figure 3:* GWAS analysis of SSOE in UKB
- *Supplementary Figure 4:* XWAS of the All-PA-Leisure phenotype for EUR MVP data
- *Supplementary Figure 5:* EUR All-PA-Leisure+SSOE meta-analysis
- *Supplementary Figure 6:* MAGMA Tissue Expression Analysis with specific tissue types for EUR All-PA-Leisure+SSOE meta-analysis
- *Supplementary Figure 7:* MAGMA Tissue Expression Analysis with general tissue types for EUR All-PA-Leisure+SSOE meta-analysis
- *Supplementary Figure 8:* MAGMA Tissue Expression Analysis with specific tissue types for cross-ancestry All-PA-Leisure+SSOE meta-analysis
- *Supplementary Figure 9:* MAGMA Tissue Expression Analysis with general tissue types for cross-ancestry All-PA-Leisure+SSOE meta-analysis.
- *Supplementary Figure 10:* Genetic correlation between EUR All-PA-Leisure+SSOE meta-analysis after conditioning on income, and other traits of interest
- *Supplementary Figure 11:* EUR MVP GWAS analysis of the All-PA-Leisure&Home phenotype
- *Supplementary Figure 12:* EUR MVP GWAS analysis of the All-PA-Leisure&Home&Work phenotype
- *Supplementary Figure 13: Manhattan plot of Vig-PA-Leisure*
- *Supplementary Figure 14: Manhattan plot of Vig-PA-Work time*

- *Supplementary Figure 15: Manhattan plot of Vig-PA-Home time GWAS*
- *Supplementary Figure 16: XWAS of Vig-PA-Leisure phenotype of EUR MVP data*
- *Supplementary Figure 17: MAGMA Tissue Expression Analysis with specific tissue types for Vig-PA-Leisure GWAS*
- *Supplementary Figure 18: Genetic correlation between each pair of vigorous PA (Vig-PA-Leisure, Vig-PA-Work, Vig-PA-Home) after conditioning on income, and other traits of interest*
- *Supplementary Figure 19:* Distribution of All-PA-Leisure score and its Yeo-Johnson transformation for MVP EUR data.
- *Supplementary Figure 20:* Quantile-quantile (QQ) plot, All-PA-Leisure phenotype in MVP EUR

VA Million Veteran Program core acknowledgement

Supplementary Methods

MVP datasets

The U.S. Department of Veterans Affairs (VA) Million Veteran Program (MVP) is one of the largest and most diverse biobanks in the world with genetic and electronic health record (EHR) data available.[1,2] Ethical approval of the MVP study was given by The Central VA Institutional Review Board (IRB) and site-specific IRBs. All relevant ethical regulations for work with human subjects were followed in the conduct of the study, and informed consent was obtained from all participants.

Participants provided a blood sample for genomic analyses, granted access to medical records, and many agreed to complete two questionnaires, the MVP Baseline and Lifestyle Surveys. PA information used in this study was collected from the MVP Lifestyle Survey, and data were subdivided according to context (leisure, work, and home), intensity (vigorous, moderate, and light), and frequency (Supplementary Fig. 1). The three levels of activity were defined as following:

1) *Vigorous*: activities that cause your heart to beat rapidly and you work up a good sweat and are breathing heavily; performed at least 10 minutes at a time;
2) *Moderate*: activities that cause your heart rate to increase slightly and you typically work up a sweat, but are not physically exhausting; performed for at least 10 minutes at a time;
3) *Light*: activities that require little physical effort".

The levels of activity were considered together with frequency information, which could be "daily", "several times/week", "once/week", "several times/month", "once/month or less", or "never", when participants answered to three questions regarding the place where PA was effected. The questions asked to participants in the MVP Lifestyle Survey were the following:

During the job time: *while at your job, how often do you engage in the following levels of activity?*

	Daily	Several times/ week	Once/ week	Several times/ month	Once/ month or less	Never
**Vigorous** (e.g., lifting heavy objects, long periods of rapid movements, intensive physical labor)	☐	☐	☐	☐	☐	☐
**Moderate** (e.g., extended periods of walking, operating heavy power tools)	☐	☐	☐	☐	☐	☐
**Light** (e.g., office work, driving a small vehicle, sales)	☐	☐	☐	☐	☐	☐

During at home time: *while performing chores in and around your home, how often do you engage in the following levels of activity?*

	Daily	Several times/ week	Once/ week	Several times/ month	Once/ month or less	Never
**Vigorous** (e.g., lifting heavy objects, digging in the garden)	☐	☐	☐	☐	☐	☐
**Moderate** (e.g., mowing the lawn with a push mower)	☐	☐	☐	☐	☐	☐
**Light** (e.g., light housework like dusting or laundry)	☐	☐	☐	☐	☐	☐

During leisure time: *during your leisure or free time, how often do you engage in the following levels of activity?*

	Daily	Several times/ week	Once/ week	Several times/ month	Once/ month or less	Never
**Vigorous** (e.g., competitive sports like running, swimming, or high intensity aerobics)	☐	☐	☐	☐	☐	☐
**Moderate** (e.g., low impact aerobics, or golfing without a power cart)	☐	☐	☐	☐	☐	☐
**Light** (e.g., bowling, archery, easy walking, golfing with a power cart, fishing)	☐	☐	☐	☐	☐	☐

For the All-PA phenotype, the three different levels of PA were summed considering their frequency and weighting intensity to create a total score using the following table:

	Daily	Several/week	Once/week	Several/month	Once/month	Never
Vigorous	15	12	9	6	3	0
Moderate	10	8	6	4	2	0
Light	5	4	3	2	1	0

After MVP genotyping, which was performed using a customized Affymetrix Axiom Biobank Array and quality control,[3] EAGLE2[4] was used for phasing chromosomes and Minimac3 was used for imputation,[5] using the 1000 Genomes Project reference panel, phase 3, version 5.[6] Populations were defined using principal component analysis.[1]

For All-PA during leisure time (All-PA-Leisure), we ran independent GWAS for each of three ancestries (EUR; African (AFR); and Admixed American (Latino) (AMR)) (Supplementary Fig. 1). For EUR, we also ran GWAS for vigorous PA during leisure time (Vig-PA-Leisure), work time (Vig-PA-Work), and home time (Vig-PA-Home). We did not have enough power to run GWAS for vigorous PA for AFR and AMR ancestries.

In the quality control procedure for GWAS conducted using PLINK 2.0[7], we removed variants with imputation quality scores<0.6, Hardy-Weinberg equilibrium *p*-value $< 5\times10^{-5}$, minor allele frequency<0.01, missing call rates for variants>0.1, and missing call rates for samples>0.1. Data were aligned to the GRCh37 reference genome. Considering EUR ancestry, after filtering we had 6660206 variants for All-PA-Leisure phenotype, and 6548052, 6536056, 6537158 variants for Vig-PA-Leisure, Vig-PA-Home, and Vig-PA-Work, respectively. For AFR and AMR ancestries, we kept 11984943 and 7969366 variants, respectively (All-PA-Leisure phenotype).

To remove related individuals, we used a threshold of 0.0884 for the kinship coefficients calculated by KING,[8] resulting in removal of individuals with minimum a second-degree relationship. Then, we implemented an algorithm to optimize keeping the maximum number of individuals with the highest score (for All-PA-Leisure phenotype) or with the highest frequency (for the vigorous activity phenotypes). If two individuals had the same score (or frequency), we would have removed the one with the highest number of relationships. For EUR, we finally had 189,812 individuals for All-PA-Leisure phenotype, and 201,050 for Vig-PA-Leisure, 203,430 for Vig-PA-Home, and 171,278 for Vig-PA-Work. For AFR and AMR ancestries, we kept 27,044 and 10,263 individuals, respectively (All-PA-Leisure phenotype). We ran GWAS analysis using a linear regression model implemented in PLINK 2.0, using sex, age, and the first ten principal components as covariates.

For All-PA-Leisure&Home phenotype, and for All-PA-Leisure&Home&Work, we ran the same QC as described in the main methods, using EUR ancestry. After filtering we had 6678873 variants for All-PA-Leisure&Home, and 6761070 variants for All-PA-Leisure&Home&Work. For the sample size, where we excluded individuals without full information of their PA, we finally had 181,317 individuals for All-PA-Leisure&Home, and 146,929 All-PA-Leisure&Home&Work.

MVP All-PA-Leisure GWAS validation

The All-PA-Leisure phenotype does not demonstrate a normal distribution. We therefore evaluated whether this could have influenced our GWAS results, including the following analyses. We checked the MAF from the GWAS of EUR All-PA-Leisure of the significant variants, since the normality assumption is most likely to affect results for rare variants.[9,10] We created a score transformation to reproduce a normal distribution of MVP All-PA-Leisure (see

subsection **MVP All-PA-Leisure score transformation below**). We calculated the intercept and attenuation ratios using LDSC.[11]

MVP All-PA-Leisure score transformation

To normalize the All-PA-Leisure score for EUR data in MVP, we transformed it with the Yeo-Johnson transformation after removing individuals with All-PA-Leisure score equal to zero. Yeo-Johnson is a transformation acting on a continuous variable to make it more normally distributed (distributions of the original All-PA-Leisure score and the Yeo-Johnson transformation are plotted in Supplementary Fig. 19). Thus, we ran a new GWAS using this transformed score as phenotype. The genetic correlation between the transformed All-PA-Leisure trait and the original All-PA-Leisure was r_g=0.987±0.006 with p-value<10^{-300}. The high correlation between results from the original analysis and the analysis using the transformed phenotypes confirms the validity of using the original All-PA-Leisure phenotype for our subsequent analyses.

Traits studied for genetic correlation with PA

We estimated the genetic correlation of All-PA-Leisure+SSOE with traits related to cardiovascular disease, respiratory system disease, anthropometric traits, metabolic traits, biological age measures, lifestyle traits, cognitive traits, substance use traits, food consumption, and additional disease traits (Supplementary Table 41). For these traits, we downloaded EUR-ancestry summary statistics, except for COVID-19, for which the statistics without MVP (and 23andME) data were available only for all ancestries. EUR COVID-19 data represented 91% of the total sample size.

Cardiovascular diseases included abdominal and thoracic aortic aneurysm,[12] coronary artery disease,[13] heart failure (a clinical syndrome defined by fluid congestion and exercise intolerance due to cardiac dysfunction),[14] and stroke (which includes ischemic stroke and intracerebral hemorrhage).

Respiratory system diseases included asthma and two traits related to COVID-19, one considering infected individuals versus population (COVID-19), and the other comparing hospitalized cases versus population (COVID-19 hospitalization).[15]

Anthropometric traits included height, bone mineral density,[16] and body mass index (BMI).[17]
Metabolic traits included high-density lipoprotein (HDL) cholesterol, type 1 diabetes,[18] type 2 diabetes,[19] and triglycerides.

Biological age measures included Biological Age Acceleration,[20] Phenotypic Age Acceleration,[20] and parental survival.[21] Biological Age Acceleration and Phenotypic Age Acceleration represent the expected age within the population that corresponds to a person's estimated mortality risk after accounting for biological or chronological age.[20] Biological Age Acceleration and Phenotypic Age Acceleration were estimated respectively by the residual of PhenoAge or BioAge after subtracting the effect of chronological age using a linear regression model. PhenoAge and BioAge are two validated biological age predictors.[22,23] Using biomarker data from the National Health and Nutrition Examination Survey III in the United States, PhenoAge was trained for mortality as a surrogate of biological age, and BioAge was trained for the biological age surrogate of chronological age. PhenoAge is a function of chronological age, albumin, creatinine, C-reactive protein, alkaline phosphatase, glucose, lymphocyte percentage, mean corpuscular volume, red blood cell distribution width, and white blood cell count. BioAge is a function of chronological age, albumin, creatinine, C-reactive protein, alkaline phosphatase, glycated hemoglobin, systolic blood pressure, and total cholesterol.

Lifestyle traits included liking of PA,[24] household income, wellbeing,[25] neuroticism, and leisure screen time.[26]

Cognitive traits included educational attainment,[27] executive functioning,[28] Alzheimer disease,[29] and Parkinson's disease.[30]

Substance use traits included cigarettes per day[31] and drinks per week.[31]

Food consumption included the behaviors of eating fruit, olive oil, vegetables, and cheese.
Additional disease traits included inflammatory bowel disease,[32] gastroesophageal reflux disease,[33] lung cancer,[34] osteoarthritis, breast cancer,[35] colorectal cancer,[34] oral cancer,[34] and multiple sclerosis.[36]

A report of the study accession is provided in Supplementary Table 41. Data from the Neale lab were from UKB and downloaded from the website http://www.nealelab.is/uk-biobank/.

Multi-trait conditional and joint analysis

Multi-trait conditional and joint analysis (*mtCOJO)* was performed to evaluate possible confounding of socio-economic status on All-PA-Leisure+SSOE meta-analysis and in the different kinds of the vigorous physical activities using mtCOJO utility of GCTA.[37] Socio-economic status was represented with income GWAS summary statistics reported by

the Neale lab (https://www.nealelab.is/uk-biobank). The conditional analysis was performed individually on All-PA-Leisure+SSOE meta-analysis and for each of the three PA traits defined in our work: Vig-PA-Leisure, Vig-PA-Home, and Vig-PA-Work. The respective PA GWAS summary statistic was used as the target trait and conditioned on the income (covariate trait).

Multivariable Mendelian Randomization
We also used TwoSampleMR to run multivariable MR (MVMR) analyses of All-PA-Leisure phenotype as exposure, to understand the influence that BMI could have as a confounder, setting it as exposure. As outcomes, we chose previously-significant MR traits which had health importance: abdominal aortic aneurysm, HDL-C, type 2 diabetes, triglycerides, phenotypic age acceleration, gastroesophageal reflux disease, and osteoarthritis. We excluded heart failure from the analyses because of the problem of overlapping cohorts. To avoid this problem, for COVID-19 hospitalization we used data from the COVID-19 host genetics initiative without 23andME and MVP data. For the MVMR analyses, we applied a multiple testing (*p*-value=0.0063) because of the 8 outcomes.

Supplementary Results

MVP All-PA-Leisure GWAS validation
As noted, the distribution of All-PA-Leisure phenotype does not demonstrate a normal distribution. We evaluated how this might have affected our results.
1. We analyzed only common variants, whereas violation of the normality assumption is most likely to affect results for rare variants.[9,10] We did not observe any statistically significant variants at the lowest MAF=0.01. The significant variant with the lowest MAF had a MAF=0.063. Thus, our significant findings lie quite far from the range where a possible effect of the lack of normality would be expected.
2. In the analysis to test whether the normality violation was likely to have affected the results presented, a GWAS analysis based on the normally transformed All-PA-Leisure phenotype, resulted in a r_g=0.987±0.006 (*p*-value<10^{-300}) with the original All-PA-Leisure GWAS ("MVP All-PA-Leisure score transformation" in Supplementary Methods). That is, the results from transformed vs. original data show close genetic similarity.
3. Additionally, we observed a lack of evidence for inflation or deflation in our data from the original MVP All-PA-Leisure GWAS as measured by the intercept (1.034±0.008) and attenuation ratio of the LD score regression (0.094±0.023). Since there was a lack of inflation, this is more evidence that the phenotype distribution did not lead to false positive results. These results provided by LD score regression suggest also that the inflation observed p-value versus expected p-value seen in the QQ plot of the All-PA-Leisure trait for EUR MVP data (Supplementary Fig. 20) is caused by polygenicity and does not reflect the distribution of the phenotype All-PA-Leisure.[11]

SNP heritability and genetic correlation of PA phenotypes
In MVP data for All-PA-Leisure, SNP-based heritability (SNP-h^2) is 0.083±0.005 (standard error (SE)) in EUR, 0.034±0.017 in AFR, and 0.083±0.036 in AMR (Table 1). SNP-h^2 of SSOE from UKB is 0.061±0.003. The genetic correlation between All-PA-Leisure and SSOE (r_g=0.76±0.03; *p*-value=4.7×10^{-118}) was higher than the genetic correlation between All-PA-Leisure and the other two traits from the previous UKB report,[38] moderate-to-vigorous PA (r_g=0.25±0.04; *p*-value=4.3×10^{-11}), and vigorous PA (r_g=0.55±0.04; *p*-value=3.9×10^{-53}). Accordingly, we selected the SSOE phenotype for meta-analyses with MVP data, i.e. the All-PA-Leisure+SSOE meta-analysis (see *Methods*).

TWAS and fine-mapping analyses of All-PA-Leisure+SSOE meta-analyses
We performed a TWAS with FUSION to identify gene associations between EUR All-PA-Leisure+SSOE meta-analysis and tissue-specific gene expression data.[39] We used EUR All-PA-Leisure+SSOE meta-analysis because of the use of EUR linkage disequilibrium (LD) reference data in FUSION. We discovered 80 independent significant genes (Supplementary Table 9). Running FOCUS fine-mapping analyses following common posterior inclusion probability (PIP) thresholds,[40] within the 90%-credible set we found 12 with PIP ≥ 0.9 and 19 genes with PIP ≥ 0.7, respectively (Supplementary Table 10). Fine-mapping analysis at the SNP level (PolyFun) provided 53 SNPs with PIP≥0.95, belonging to 37 genes (Supplementary Table 11).

Gene-based association analyses, gene-set analyses, and functional enrichment of cross-ancestry All-PA-Leisure+SSOE meta-analysis
From MAGMA gene-based test we obtained 107 significant genes from 19121 protein coding genes (*p*-value=2.6×10^{-6}) (Supplementary Table 15). Like for the EUR meta-analysis, *CADM2* was the strongest significant gene (*p*-value=1.1×10^{-18}). The second most significant gene-based association was still on chromosome 3: *CAMKV* (*p*-value=6.6×10^{-15}).

Eight significant gene ontology (GO) terms resulted from the MAGMA gene-set analysis (Supplementary Table 16), with three shared terms between EUR ancestry and cross-ancestry: structural constituent of presynapse, presynaptic active zone organization, and maintenance of presynaptic active zone structure.

MAGMA Tissue Expression Analysis showed four significant enrichments in the brain tissues, with still the two most significant represented in the cerebellar hemisphere and in the cerebellum (Supplementary Fig. 8, 9).

Mitochondrial genome association analysis of EUR All-PA**-Leisure**

Mitochondria-SNV association analysis resulted only in a nominally significant marker (rs199951903; *p*-value=0.024; MAF=0.0034) mapping to gene *CYTB*, which encodes Cytochrome b, a component of the mitochondrial electron transport chain. Gene-based association analysis on the mitochondrial genome did not identify any significant results.

Genetic correlation of EUR All-PA-Leisure+SSOE with traits of interest

Negative genetic correlation was observed with the disease traits, most of anthropometric and metabolic traits (including body mass index (BMI)), the two biological age measures, neuroticism, leisure screen time, and also cigarettes per day. Positive genetic correlations with All-PA-Leisure+SSOE were founded for liking of PA, household income, educational attainment, HDL cholesterol, wellbeing, height, drinks per week, executive functioning, and the analyzed consumed foods: fruit, olive oil, vegetables, and cheese. Genetic correlations were also calculated with other cancer types (breast, colorectal, and oral cancer), Parkinson's disease, and multiple sclerosis, but none were significant (Supplementary Table 19).

PheWAS of All-PA-Leisure+SSOE

Phenome-wide association study (PheWAS) in BioVu using a polygenic score for PA derived from EUR All-PA-Leisure+SSOE meta-analysis, revealed significant association with 159 traits (Fig. 2A, Supplementary Table 21). The strongest PheWAS results included negative associations with diabetes mellitus (odds ratio (OR)=0.868; *p*-value=5.89×10^{-36}), and chronic airway obstruction (OR=0.84; *p*-value=4.22×10^{-32}). We also found significant negative associations for traits also analyzed in the genetic correlation analyses: coronary artery disease (ischemic heart disease) (OR=0.89; *p*-value=1.02×10^{-21}), gastroesophageal reflux disease (OR=0.93; *p*-value=5.07×10^{-13}), cancer of bronchus or lung (OR=0.90; *p*-value=5.47×10^{-7}), and asthma (OR=0.94; *p*-value=1.19×10^{-5}). We also found a negative correlation with obesity (OR=0.90; *p*-value=2.38×10^{-17}), and several phenotypes related to heart failure: diastolic heart failure (OR=0.88; *p*-value=1.76×10^{-9}), systolic or combined heart failure (OR=0.89; *p*-value=1.96×10^{-9}), and heart failure (OR=0.90; *p*-value=1.88×10^{-5}). The most significant positive associations were with benign neoplasm of skin (OR=1.17; *p*-value=2.41×10^{-19}) and allergic rhinitis (OR=1.12; *p*-value=3.23×10^{-19}).

Mendelian Randomization analyses of All-PA-Leisure

The MR analyses with the two p-value thresholds also differed for several relationships detected for *p*-value threshold $< 1 \times 10^{-5}$ but not 5×10^{-8}: for PA as exposure with abdominal aortic aneurysm, heart failure, height, phenotypic age acceleration, and wellbeing; for PA as outcome with HDL-C, type 2 diabetes, parental survival, neuroticism, and cigarettes per day. We tested horizontal pleiotropy for the significant causal relationships related to health traits, without finding influences (Supplementary Table 25).

Genes detected by gene-based approaches

Gene-based approaches highlighted 2 genes (*CADM2* and *AKAP10*) shared by 4 approaches, and 8 genes (*RGS12*, *NCP1*, *GATAD2A*, *DLAT*, *CYP46A1*, *ANK3*, *AFF3*, *ACTN1*) shared by 3 approaches.

Four of the 8 genes shared by 3 approaches, *NCP1*, *DLAT*, *ANK3, ACTN1* constitute novel evidence of genes having a role in leisure time PA. *GATAD2A* had a variant previously found associated to SSOE[38], and with a contribution to enrichment in the association with significantly altered expression in skeletal muscle following acute resistance and acute aerobic[26]. *RGS12* had a variant associated to LST (rs743699) but it was not found related to PA.[26] *CYP46A1* was previously found contributing on tissue enrichment analysis of visual cortex and occipital lobe for moderate to vigorous PA during leisure time based on public microarray expression data.[26] *AFF3* was mentioned for moderate to vigorous PA during leisure time in summary-based Mendelian randomization approach (SMR) with brain data.[26]

The 2 genes *CADM2* and *AKAP10*, which were shared by 4 approaches, were broadly discussed in previous works on PA[26,38].

MTAG analyses of EUR All-PA-Leisure+SSOE

We ran an MTAG analysis between the EUR meta-analysis for All-PA-Leisure+SSOE phenotype and leisure screen time, for which we used the EUR ancestry meta-analysis previously reported, which included a total of 526,725 individuals from self-reported data.[26] For the All-PA-Leisure+SSOE trait, our MTAG analysis provided 74 lead SNPs and 64 genomic associated loci (Supplementary Table 12). 36 of these lead SNPs were previously not found significant associated to PA, and 59 were previously not found to be significantly associated to leisure screen time. However, only 14 of the 67 previously-identified lead SNPs in the EUR meta-analysis were also significant variants in the MTAG analysis.

We also ran MTAG between the EUR meta-analysis for All-PA-Leisure+SSOE phenotype and liking of PA, given their highest positive genetic correlation (r_g=0.77±0.02; p-value=1.3×10^{-210}, Z_{score}=30.97). Liking of PA is an overall measure derived from 5 PA-liking items in UKB including 151,347 individuals: going to the gym, working up a sweat, exercising with others, exercising alone, and bicycling.[24] For the PA trait, this analysis provided 59 lead SNPs and 50 genomic associated loci (Supplementary Table 13). 21 of these lead SNPs were not found previously associated to PA, but only 13 of the previously 67 lead SNPs in the EUR meta-analysis were also maintained significant variants in this MTAG analysis.

The two MTAG analyses, All-PA-Leisure+SSOE with leisure screen time and All-PA-Leisure+SSOE with liking of PA, shared 29 lead SNPs.

Kinds of vigorous PA and their genetic interrelationships
Vig-PA-Leisure GWAS identified 8 lead SNPs and 7 genomic associated loci (Supplementary Fig. 13, Supplementary Table 28). Vig-PA-Work GWAS identified one significant intergenic variant (rs12968236 on chromosome 18) (Supplementary Fig. 14). Vig-PA-Home GWAS did not identify any significant variants (Supplementary Fig. 15).

Thus, we considered Vig-PA-Leisure (SNP-h^2 Z_{score}=16.34), which had apparent higher power compared to Vig-PA-Work (SNP-h^2 Z_{score}=5.83) and Vig-PA-Home (SNP-h^2 Z_{score}=13.65). The highest peak of the Manhattan plot was the variant *SPATS2**rs191602006 on chromosome 12 (p-value=1.4×10^{-10}), which was also significant in the both previously seen All-PA-Leisure+SSOE meta-analyses of EUR ancestry and cross-ancestry. A sex stratified analysis of the X chromosome conducted on the Vig-PA-Leisure phenotype did not show any significant associated variant (Supplementary Fig. 16).

The gene-based test computed by MAGMA identified 19 significant genes from 18,713 protein coding genes (GWS defined as p-value=2.672×10^{-6}) (Supplementary Table 29). As in the All-PA-Leisure+SSOE meta-analyses, *CADM2* was the strongest significant gene (p-value=2.0×10^{-21}). We did not obtain any significant GO term from MAGMA gene-set analysis. MAGMA Tissue Expression Analysis showed two significant enrichments in the brain tissues: cerebellum and cerebellar hemisphere (Supplementary Fig. 17). FUMA cell type enrichment analysis did not identify significant human cell types across the datasets (see subsection *Single-cell expression* in *Methods*).

The TWAS performed to link the Vig-PA-Leisure phenotype GWAS to tissue-specific gene expression data identified 5 independent significant genes (Supplementary Table 31). From the fine-mapping of causal gene sets results, within the 90%-credible set we found one gene (*SLC39A9*) having PIP≥0.9 and two more genes (*IQCH*, *BDH2*) with PIP≥0.7 (Supplementary Table 32). Fine-mapping analysis at the SNP-level provided 2 SNPs with PIP≥0.95 (Supplementary Table 33). One of them, *SLC39A8**rs13107325, was also a lead SNP and a genomic risk locus in the MTAG analysis between EUR All-PA-Leisure+SSOE meta-analysis and liking of PA (Supplementary Table 13).

Functional enrichment analysis using the Vig-PA-Leisure phenotype resulted in a significant term from the protein database CORUM:[41] the protein complex MAP2K5-PRKCI-SQSTM1 (Supplementary Table 34).

Mitochondria-SNV association analysis resulted in 5 nominally significant markers (Supplementary Table 35), with rs199951903 having the lowest p-value (p-value=0.0084; MAF=0.0034). rs199951903 was also the only nominally significant marker founded in mitochondria-SNV association analysis for All-PA-Leisure (p=0.024; MAF=0.0034). Gene-based association analysis found 2 nominal significant associated genes: *MT-TV* (p-value=0.021, one locus) and *COX3* (p-value=0.031, three loci).

Multi-trait conditional and joint analysis of vigorous PA traits
We ran conditional analysis for the vigorous PA traits conditioning on income. Compared to previous results without conditioning on income, we only found a significant difference in the heritability of Vig-PA-Leisure (Supplementary Table 39). Vig-PA-Work and Vig-PA-Home did not show a significant difference in the heritability, as the genetic correlation between each pair of traits (Supplementary Table 39).

We calculated the genetic correlations between Vig-PA-Leisure, Vig-PA-Work, and Vig-PA-Home individually with the previously analyzed traits of interest, excluding income. Significant statistical differences between Vig-PA-Leisure and Vig-PA-Work were observed for 6 traits, between Vig-PA-Leisure and Vig-PA-Home for 8 traits, and between Vig-PA-Home and Vig-PA-Work for 1 trait (Supplementary Fig. 18).

Supplementary Discussion

The second most significant variant of the two meta-analyses, *MST1R**rs3733134 (EUR meta-analysis p-value=1.4×10^{-17}; cross-ancestry meta-analysis p-value=6.9×10^{-18}), encoding macrophage stimulating 1 receptor, was also significant in the GWAS of Vig-PA-Leisure (p-value=2.5×10^{-8}). Its protein product plays a role in immune response and in the development of the epithelium, brain, and neuroendocrine tissues[42]. The third most significant variant was *EXD2**rs4899292 (EUR meta-analysis p-value=1.7×10^{-16}; cross-ancestry meta-analysis p-value=3.8×10^{-16}). *EXD2* (encoding exonuclease 3'-5' domain containing 2) is involved in DNA replication.[43] Other strong significant associations included genes *LONRF2* (encoding LON peptidase N-terminal domain and ring finger 2) and *SLC39A8*, involved in the binding activity and transportation of metal ions, and *NPC1* (encoding NPC intracellular cholesterol transporter 1), involved in cholesterol transport. MR analyses showed bidirectional causality between PA and HDL-C. For the genetic liability to HDL-C causing higher PA values, a possible explanation could be that genetic liability to HDL-C tags another pleiotropic trait, such as nutritional choices. *LONRF2* and *SLC39A8* protein products influence processes involving metal ion binding and transport, respectively. Metal ions have key roles in metabolism, energy production, muscle contraction, and nerve signaling, all crucial for PA. *SLC39A8* was previously associated to several psychiatric disorders, including problematic alcohol use,[44] schizophrenia,[45] and opioid use disorder.[46] *C18orf8*, also known as *RMC1*, mapped to the second most significant peak in the gene-based test of the cross-ancestry meta-analysis, and is associated with type 2 diabetes and BMI.[47-49]

References

1. Gaziano, J.M., *et al.* Million Veteran Program: A mega-biobank to study genetic influences on health and disease. *J Clin Epidemiol* **70**, 214-223 (2016).
2. Harrington, K.M., *et al.* Gender Differences in Demographic and Health Characteristics of the Million Veteran Program Cohort. *Womens Health Issues* **29 Suppl 1**, S56-S66 (2019).
3. Hunter-Zinck, H., *et al.* Genotyping Array Design and Data Quality Control in the Million Veteran Program. *The American Journal of Human Genetics* **106**, 535-548 (2020).
4. Loh, P.-R., *et al.* Reference-based phasing using the Haplotype Reference Consortium panel. *Nature Genetics* **48**, 1443-1448 (2016).
5. Das, S., *et al.* Next-generation genotype imputation service and methods. *Nature Genetics* **48**, 1284-1287 (2016).
6. Auton, A., *et al.* A global reference for human genetic variation. *Nature* **526**, 68-74 (2015).
7. Chang, C.C., *et al.* Second-generation PLINK: rising to the challenge of larger and richer datasets. *GigaScience* **4**(2015).
8. Manichaikul, A., *et al.* Robust relationship inference in genome-wide association studies. *Bioinformatics* **26**, 2867-2873 (2010).
9. Bůžková, P. Linear regression in genetic association studies. *PLoS One* **8**, e56976 (2013).
10. Lo, M.-T., *et al.* Genome-wide analyses for personality traits identify six genomic loci and show correlations with psychiatric disorders. *Nature Genetics* **49**, 152-156 (2017).
11. Bulik-Sullivan, B.K., *et al.* LD Score regression distinguishes confounding from polygenicity in genome-wide association studies. *Nature Genetics* **47**, 291-295 (2015).
12. Kurki, M.I., *et al.* FinnGen: Unique genetic insights from combining isolated population and national health register data. *medRxiv*, 2022.2003.2003.22271360 (2022).

13. Schunkert, H., *et al.* Large-scale association analysis identifies 13 new susceptibility loci for coronary artery disease. *Nature Genetics* **43**, 333-338 (2011).
14. Shah, S., *et al.* Genome-wide association and Mendelian randomisation analysis provide insights into the pathogenesis of heart failure. *Nat Commun* **11**, 163 (2020).
15. The, C.-H.G.I. The COVID-19 Host Genetics Initiative, a global initiative to elucidate the role of host genetic factors in susceptibility and severity of the SARS-CoV-2 virus pandemic. *European Journal of Human Genetics* **28**, 715-718 (2020).
16. Kemp, J.P., *et al.* Identification of 153 new loci associated with heel bone mineral density and functional involvement of GPC6 in osteoporosis. *Nature Genetics* **49**, 1468-1475 (2017).
17. Locke, A.E., *et al.* Genetic studies of body mass index yield new insights for obesity biology. *Nature* **518**, 197-206 (2015).
18. Chiou, J., *et al.* Interpreting type 1 diabetes risk with genetics and single-cell epigenomics. *Nature* **594**, 398-402 (2021).
19. Xue, A., *et al.* Genome-wide association analyses identify 143 risk variants and putative regulatory mechanisms for type 2 diabetes. *Nature Communications* **9**, 2941 (2018).
20. Kuo, C.-L., Pilling, L.C., Liu, Z., Atkins, J.L. & Levine, M.E. Genetic associations for two biological age measures point to distinct aging phenotypes. *Aging Cell* **20**, e13376 (2021).
21. Timmers, P.R.H.J., *et al.* Genomics of 1 million parent lifespans implicates novel pathways and common diseases and distinguishes survival chances. *eLife* **8**, e39856 (2019).
22. Levine, M.E. Modeling the rate of senescence: can estimated biological age predict mortality more accurately than chronological age? *J Gerontol A Biol Sci Med Sci* **68**, 667-674 (2013).
23. Levine, M.E., *et al.* An epigenetic biomarker of aging for lifespan and healthspan. *Aging (Albany NY)* **10**, 573-591 (2018).
24. KLIMENTIDIS, Y.C., *et al.* Genome-wide Association Study of Liking for Several Types of Physical Activity in the UK Biobank and Two Replication Cohorts. *Medicine & Science in Sports & Exercise* **54**, 1252-1260 (2022).
25. Baselmans, B.M.L., *et al.* Multivariate genome-wide analyses of the well-being spectrum. *Nature Genetics* **51**, 445-451 (2019).
26. Wang, Z., *et al.* Genome-wide association analyses of physical activity and sedentary behavior provide insights into underlying mechanisms and roles in disease prevention. *Nature Genetics* **54**, 1332-1344 (2022).
27. Okbay, A., *et al.* Polygenic prediction of educational attainment within and between families from genome-wide association analyses in 3 million individuals. *Nature Genetics* **54**, 437-449 (2022).
28. Hatoum, A.S., *et al.* Genome-wide Association Study Shows That Executive Functioning Is Influenced by GABAergic Processes and Is a Neurocognitive Genetic Correlate of Psychiatric Disorders. *Biol Psychiatry* **93**, 59-70 (2023).
29. Jansen, I.E., *et al.* Genome-wide meta-analysis identifies new loci and functional pathways influencing Alzheimer’s disease risk. *Nature Genetics* **51**, 404-413 (2019).
30. Nalls, M.A., *et al.* Identification of novel risk loci, causal insights, and heritable risk for Parkinson's disease: a meta-analysis of genome-wide association studies. *The Lancet Neurology* **18**, 1091-1102 (2019).
31. Liu, M., *et al.* Association studies of up to 1.2 million individuals yield new insights into the genetic etiology of tobacco and alcohol use. *Nature Genetics* **51**, 237-244 (2019).

32. Glanville, K.P., Coleman, J.R.I., O'Reilly, P.F., Galloway, J. & Lewis, C.M. Investigating Pleiotropy Between Depression and Autoimmune Diseases Using the UK Biobank. *Biological Psychiatry Global Open Science* **1**, 48-58 (2021).
33. An, J.*, et al.* Gastroesophageal reflux GWAS identifies risk loci that also associate with subsequent severe esophageal diseases. *Nature Communications* **10**, 4219 (2019).
34. Rashkin, S.R.*, et al.* Pan-cancer study detects genetic risk variants and shared genetic basis in two large cohorts. *Nature Communications* **11**, 4423 (2020).
35. Zhang, H.*, et al.* Genome-wide association study identifies 32 novel breast cancer susceptibility loci from overall and subtype-specific analyses. *Nature Genetics* **52**, 572-581 (2020).
36. Glanville, K.P., Coleman, J.R.I., O'Reilly, P.F., Galloway, J. & Lewis, C.M. Investigating Pleiotropy Between Depression and Autoimmune Diseases Using the UK Biobank. *Biol Psychiatry Glob Open Sci* **1**, 48-58 (2021).
37. Zhu, Z.*, et al.* Causal associations between risk factors and common diseases inferred from GWAS summary data. *Nature Communications* **9**, 224 (2018).
38. Klimentidis, Y.C.*, et al.* Genome-wide association study of habitual physical activity in over 377,000 UK Biobank participants identifies multiple variants including CADM2 and APOE. *International Journal of Obesity* **42**, 1161-1176 (2018).
39. Gusev, A.*, et al.* Integrative approaches for large-scale transcriptome-wide association studies. *Nature Genetics* **48**, 245-252 (2016).
40. Mullins, N.*, et al.* Genome-wide association study of more than 40,000 bipolar disorder cases provides new insights into the underlying biology. *Nature Genetics* **53**, 817-829 (2021).
41. Giurgiu, M.*, et al.* CORUM: the comprehensive resource of mammalian protein complexes—2019. *Nucleic Acids Research* **47**, D559-D563 (2018).
42. Gaudino, G.*, et al.* The proto-oncogene RON is involved in development of epithelial, bone and neuro-endocrine tissues. *Oncogene* **11**, 2627-2637 (1995).
43. Nieminuszczy, J.*, et al.* EXD2 Protects Stressed Replication Forks and Is Required for Cell Viability in the Absence of BRCA1/2. *Molecular Cell* **75**, 605-619.e606 (2019).
44. Zhou, H.*, et al.* Genome-wide meta-analysis of problematic alcohol use in 435,563 individuals yields insights into biology and relationships with other traits. *Nature Neuroscience* **23**, 809-818 (2020).
45. Pardiñas, A.F.*, et al.* Common schizophrenia alleles are enriched in mutation-intolerant genes and in regions under strong background selection. *Nat Genet* **50**, 381-389 (2018).
46. Deak, J.D.*, et al.* Genome-wide association study in individuals of European and African ancestry and multi-trait analysis of opioid use disorder identifies 19 independent genome-wide significant risk loci. *Molecular Psychiatry* (2022).
47. Akiyama, M.*, et al.* Genome-wide association study identifies 112 new loci for body mass index in the Japanese population. *Nat Genet* **49**, 1458-1467 (2017).
48. Pulit, S.L.*, et al.* Meta-analysis of genome-wide association studies for body fat distribution in 694 649 individuals of European ancestry. *Hum Mol Genet* **28**, 166-174 (2019).
49. Vujkovic, M.*, et al.* Discovery of 318 new risk loci for type 2 diabetes and related vascular outcomes among 1.4 million participants in a multi-ancestry meta-analysis. *Nat Genet* **52**, 680-691 (2020).

Supplementary Figures

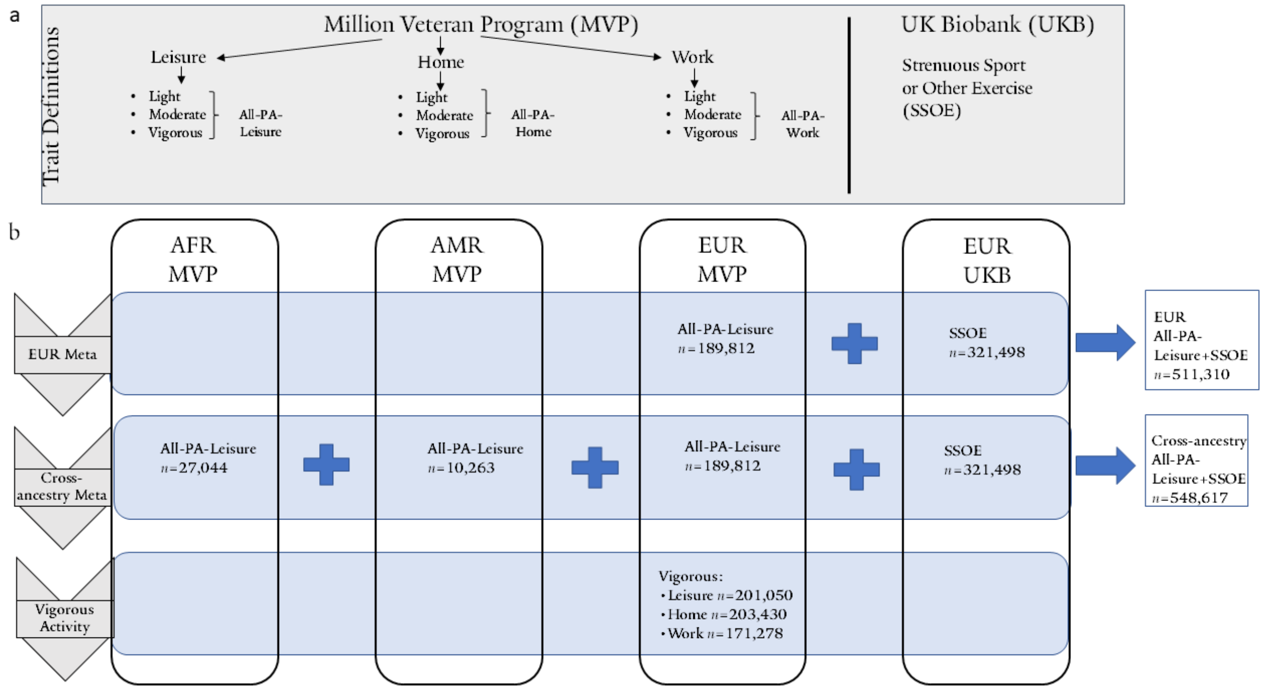

Supplementary Figure 1: **Overview of the study design. Panel a** represents a summary of the physical activity phenotypic information available in the MVP that was used in the current study, along with the UK Biobank data for Strenuous Sport or Other Exercise (SSOE)[38]. **Panel b** depicts the different levels of GWAS performed in the current study, also incorporating information on the respective cohorts and ancestries, and the overall sample size for each trait and resulting meta-analysis.

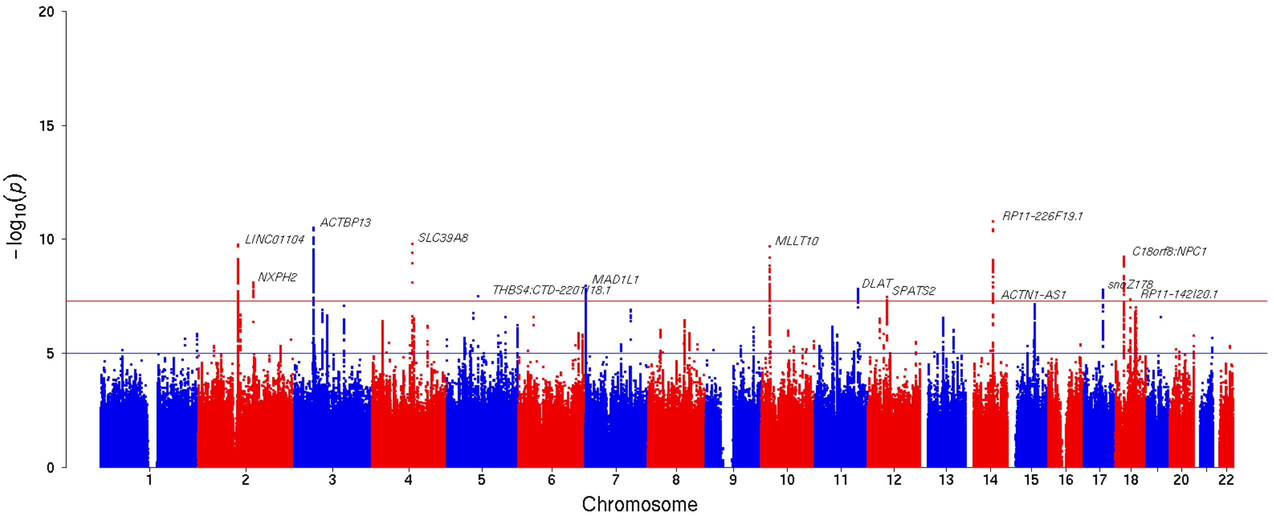

Supplementary Figure 2: **GWAS analysis of the All-PA-Leisure phenotype in MVP EUR**. Annotated genes are the nearest to each significant lead SNP. *P*-values were calculated using a linear regression model, two-sided tests. The red and blue horizontal lines indicate the genome-wide significance (p-value=5×10^{-8}) and suggestive significance (p-value=10^{-5}) levels, respectively.

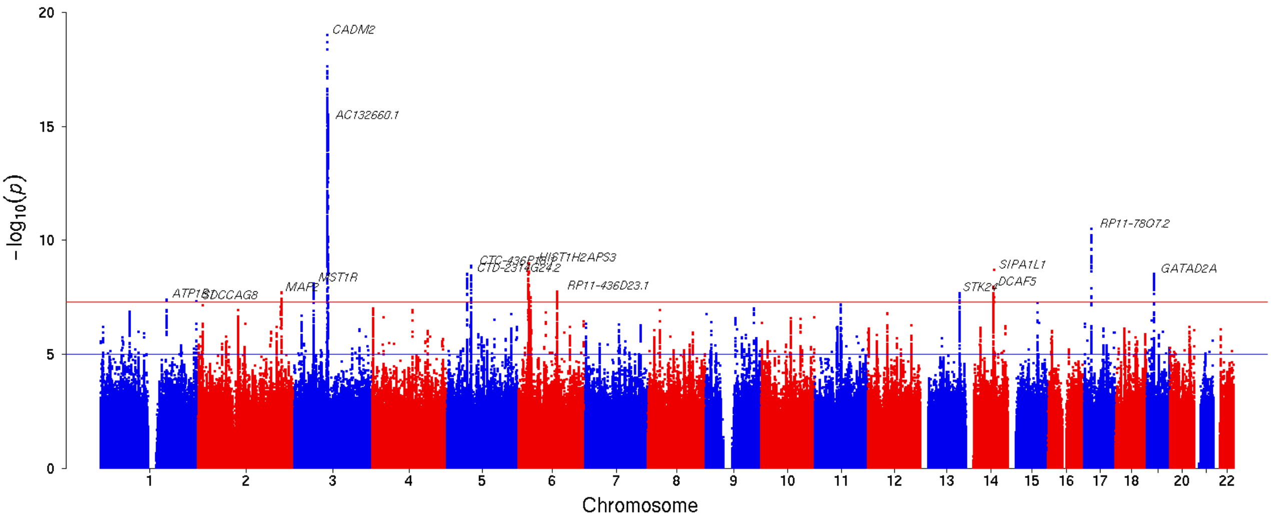

Supplementary Figure 3: **GWAS analysis of SSOE in UKB.** Annotated genes are the nearest to each significant lead SNP. *P*-values were calculated using a logistic regression model, two-sided tests. The red and blue horizontal lines indicate the genome-wide significance (*p*-value=5×10^{-8}) and suggestive significance (*p*-value=10^{-5}) levels, respectively. We added the filtering of low imputation quality <0.6 from the previous publication.[38]

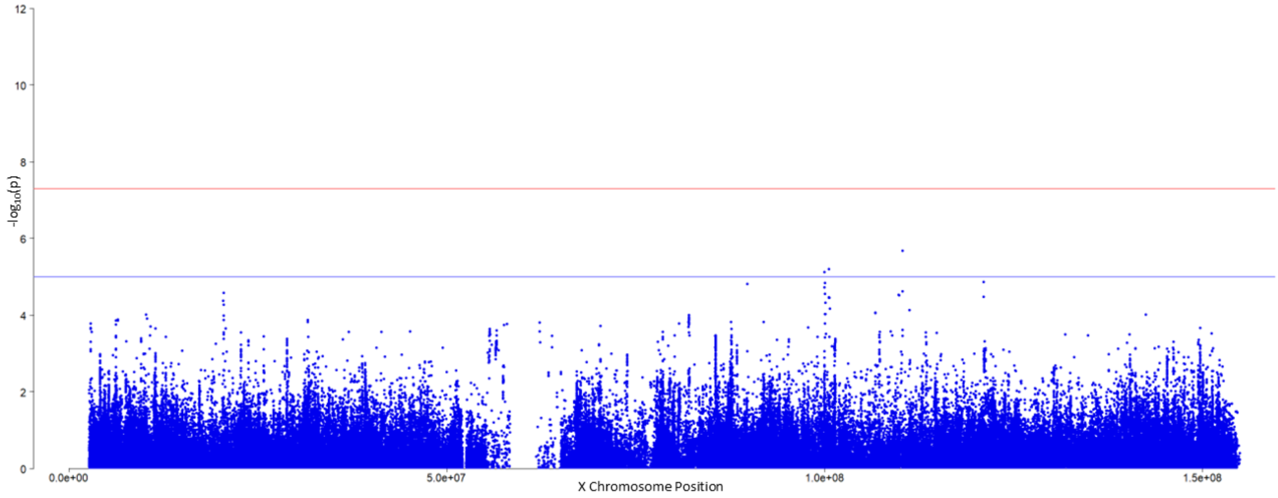

Supplementary Figure 4: **XWAS of the All-PA-Leisure phenotype for EUR MVP data.** *P*-values were calculated using a linear regression model, two-sided tests. The red and blue horizontal lines indicate the genome-wide significance (*p*-value=5×10^{-8}) and suggestive significance (*p*-value=10^{-5}) levels, respectively.

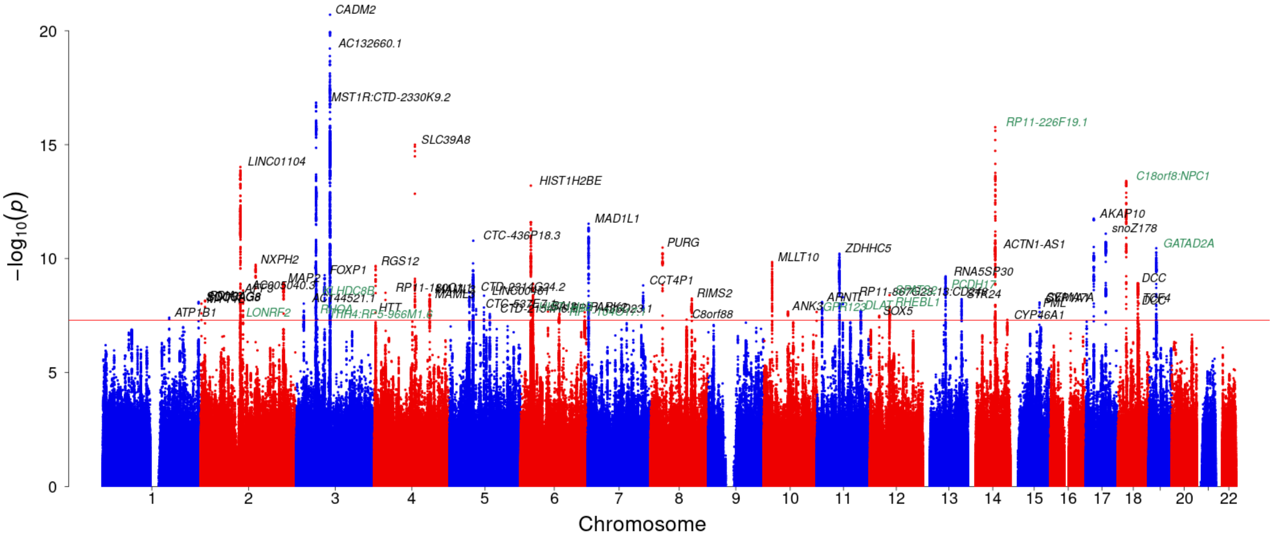

Supplementary Figure 5: **EUR All-PA-Leisure+SSOE meta-analysis.** Annotated genes are those that are closest to each significant lead SNP. Different genes compared to cross-ancestry All-PA-Leisure meta-analysis (Figure 1) are highlighted in green. *P*-values were calculated using a linear regression model, two-sided tests. The red horizontal line indicates the genome-wide significance (p-value=5×10^{-8}) level.

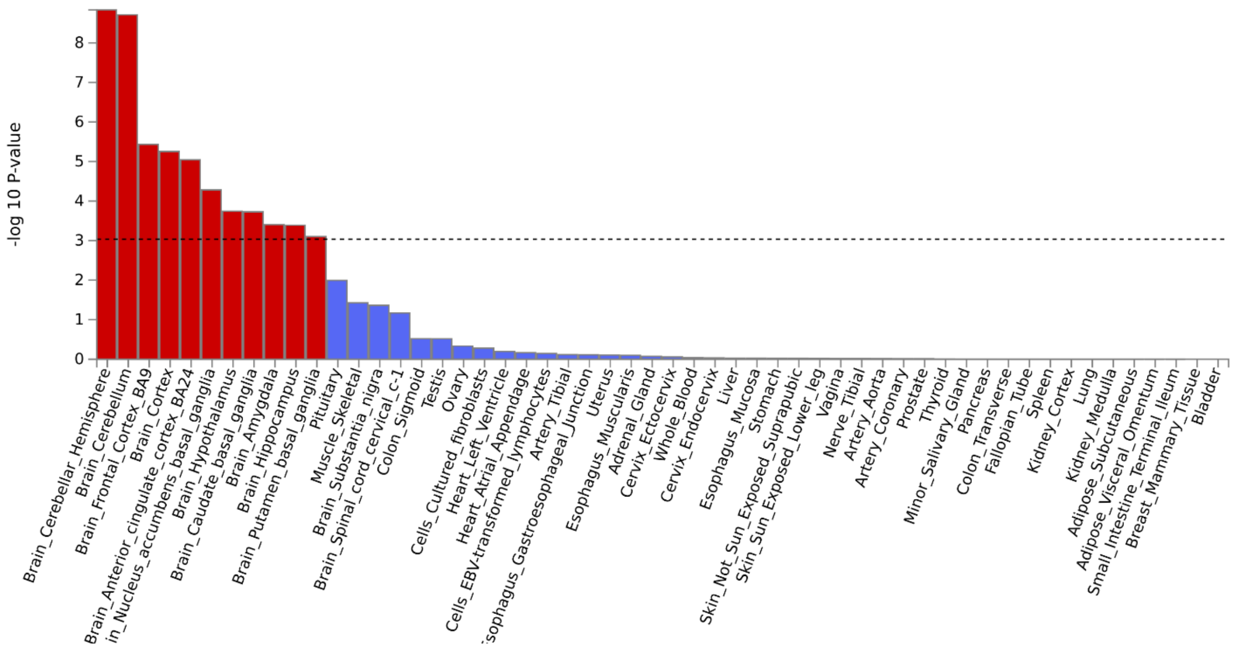

Supplementary Figure 6: **MAGMA Tissue Expression Analysis with specific tissue types for EUR All-PA-Leisure+SSOE meta-analysis.** Tests were two-sided.

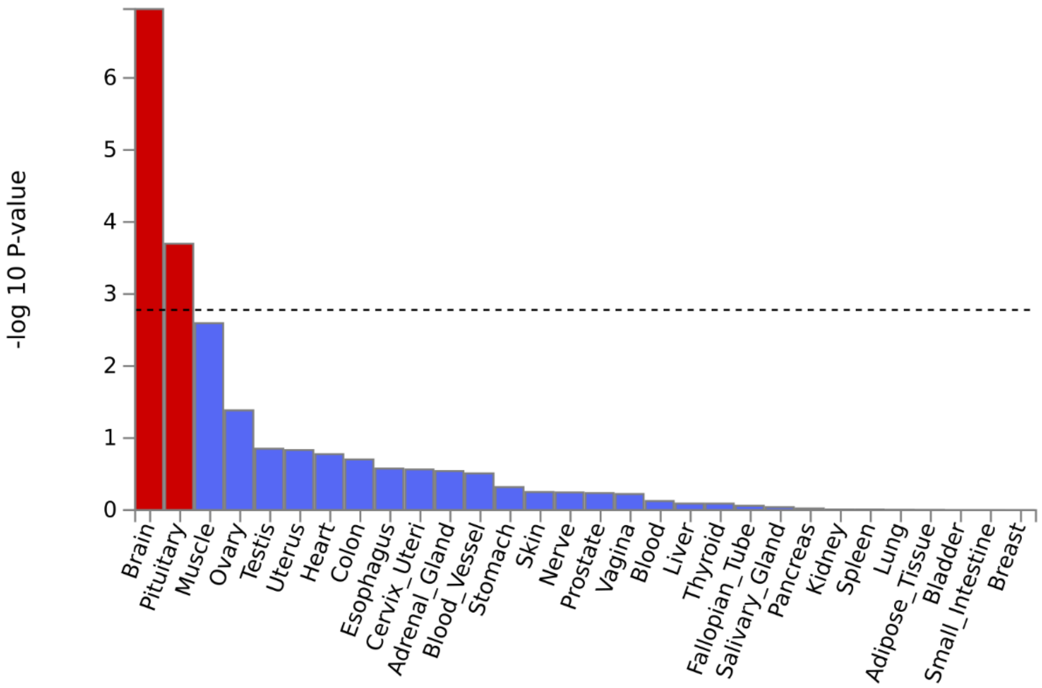

Supplementary Figure 7: **MAGMA Tissue Expression Analysis with general tissue types for EUR All-PA-Leisure+SSOE meta-analysis.** Tests were two-sided.

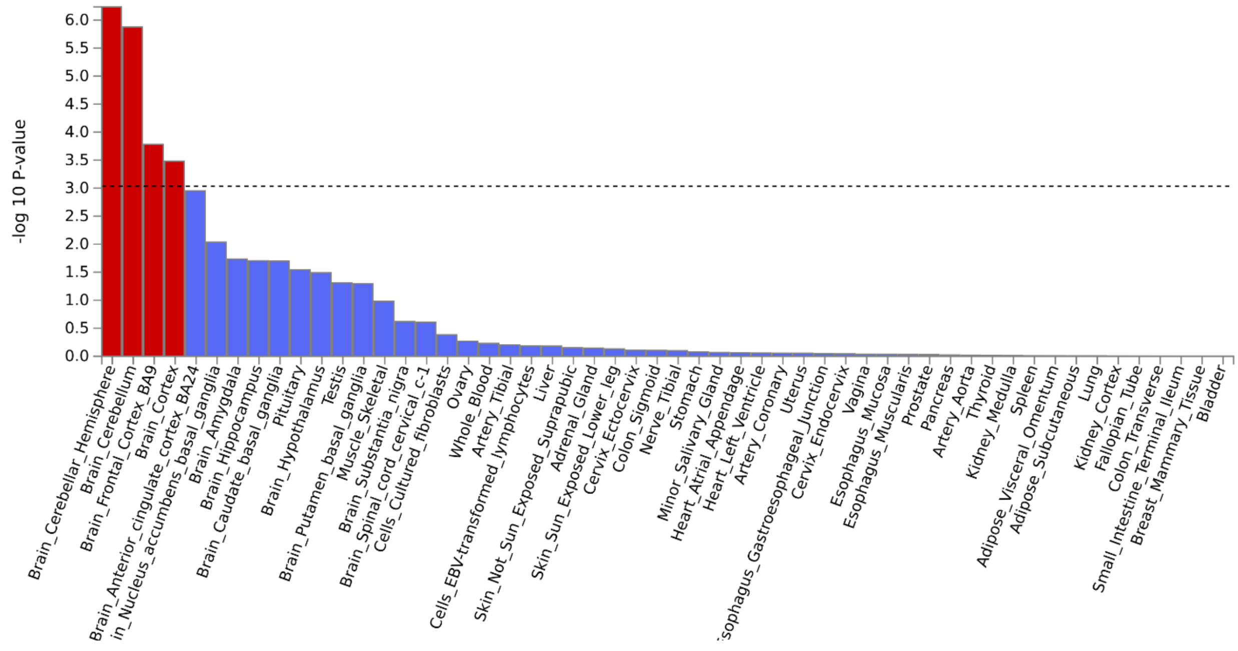

Supplementary Figure 8: **MAGMA Tissue Expression Analysis with specific tissue types for cross-ancestry All-PA-Leisure+SSOE meta-analysis.** Tests were two-sided.

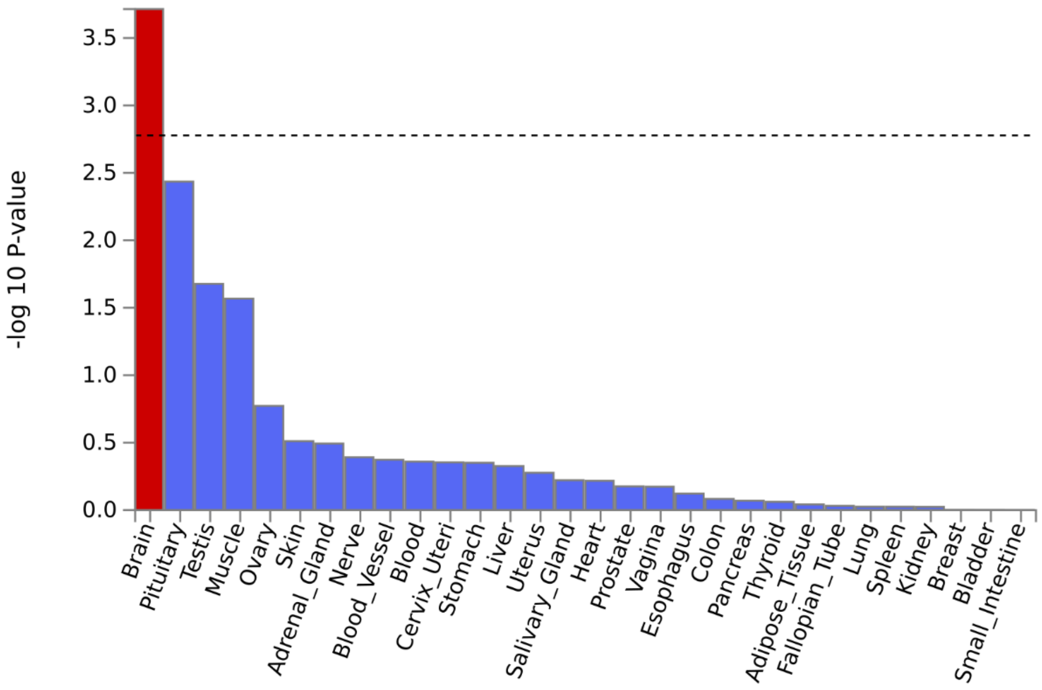

Supplementary Figure 9: **MAGMA Tissue Expression Analysis with general tissue types for cross-ancestry All-PA-Leisure+SSOE meta-analysis.** Tests were two-sided.

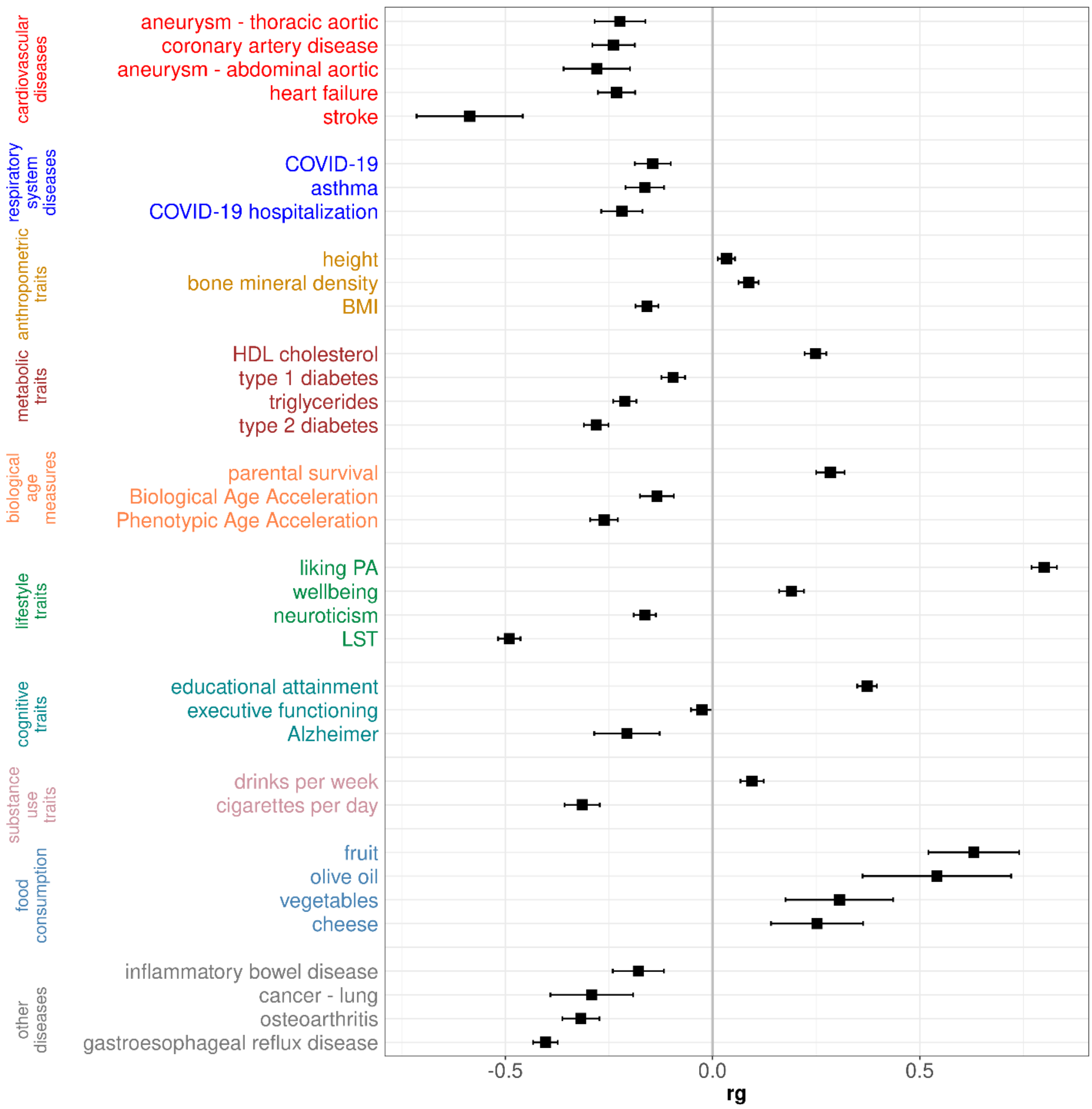

Supplementary Figure 10: **Genetic correlation between EUR All-PA-Leisure+SSOE meta-analysis after conditioning on income, and other traits of interest.** We display here the traits of interest that were significant after the Benjamini-Hochberg false discovery procedure. Tests were two-sided. Black error bars represent the standard error.

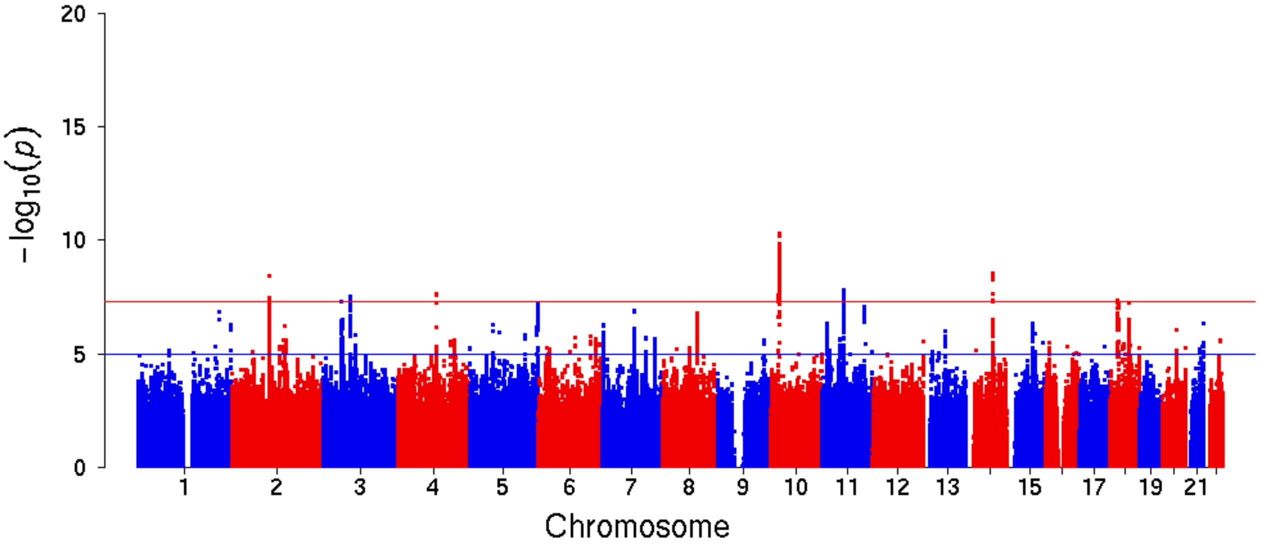

Supplementary Figure 11: **EUR MVP GWAS analysis of the All-PA-Leisure&Home phenotype.** *P*-values were calculated using a linear regression model, two-sided tests. The red and blue horizontal lines indicate the genome-wide significance (*p*-value=5×10^{-8}) and suggestive significance (*p*-value=10^{-5}) levels, respectively. We obtained 9 independent significant SNPs, 7 lead SNPs and 7 genomic associated loci.

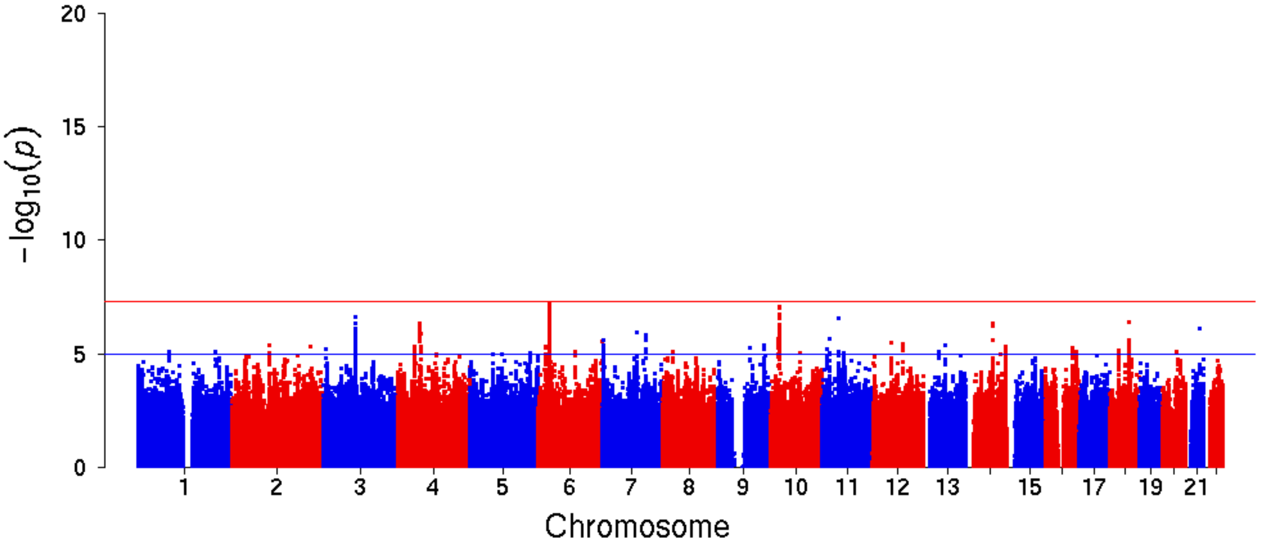

Supplementary Figure 12: **EUR MVP GWAS analysis of the All-PA-Leisure&Home&Work phenotype.** *P*-values were calculated using a linear regression model, two-sided tests. The red and blue horizontal lines indicate the genome-wide significance (*p*-value=5×10^{-8}) and suggestive significance (*p*-value=10^{-5}) levels, respectively. No significant variants were found.

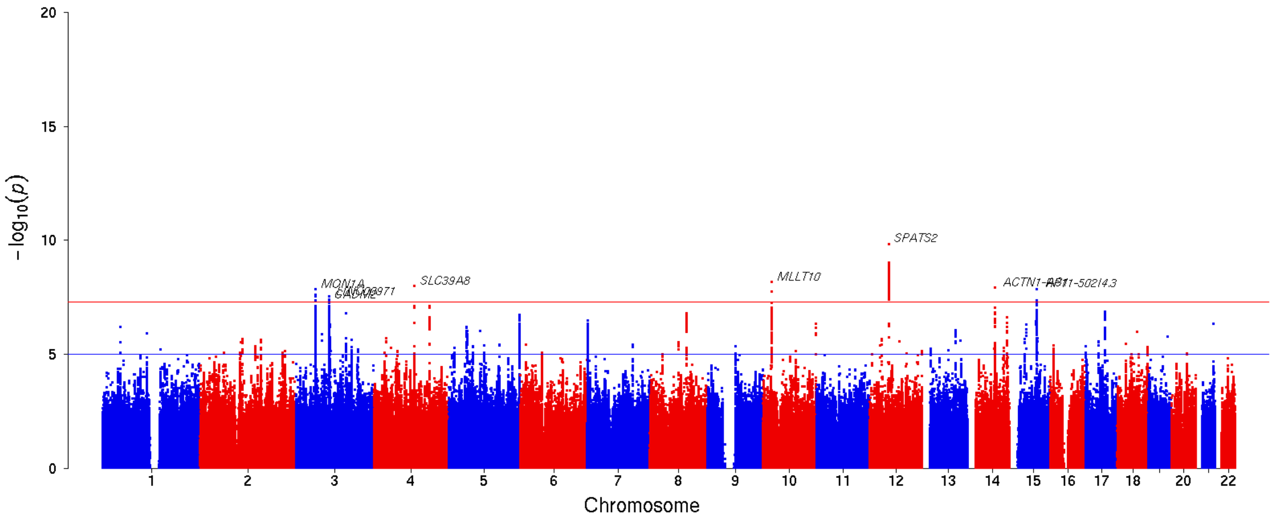

Supplementary Figure 13: **Manhattan plot of Vig-PA-Leisure.** Annotated genes are the closest to each significant lead SNP. *P*-values were calculated using a linear regression model, two-sided tests. The red and blue horizontal lines indicate the genome-wide significance (*p*-value=5×10^{-8}) and suggestive significance (*p*-value=10^{-5}) levels, respectively.

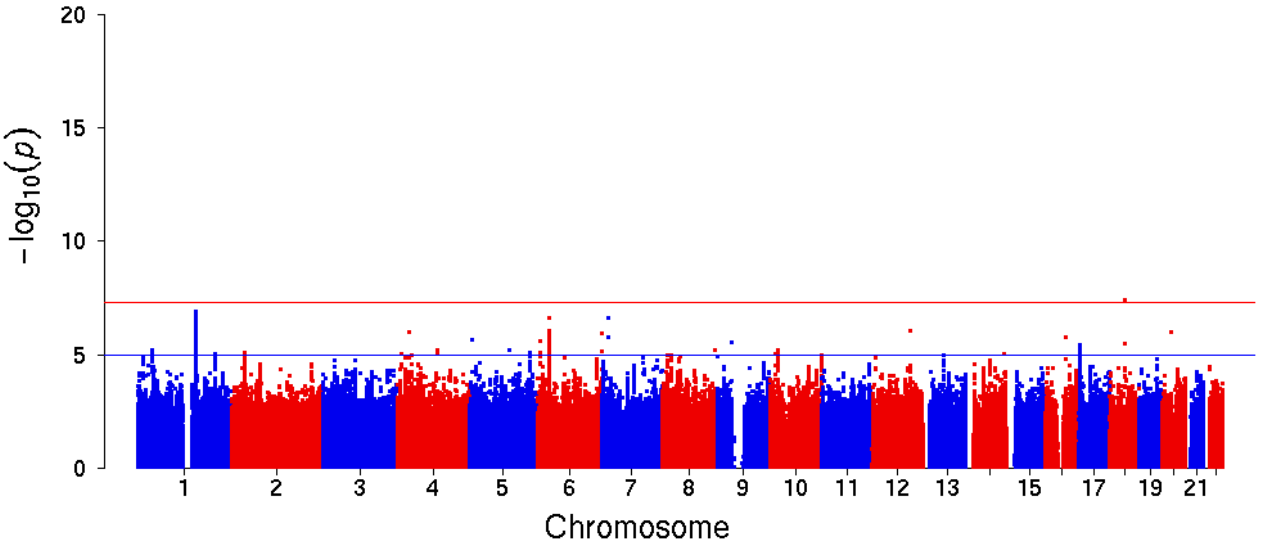

Supplementary Figure 14: **Manhattan plot of Vig-PA-Work time.** *P*-values were calculated using a linear regression model, two-sided tests. The red and blue horizontal lines indicate the genome-wide significance (*p*-value=5×10^{-8}) and suggestive significance (*p*-value=10^{-5}) levels, respectively.

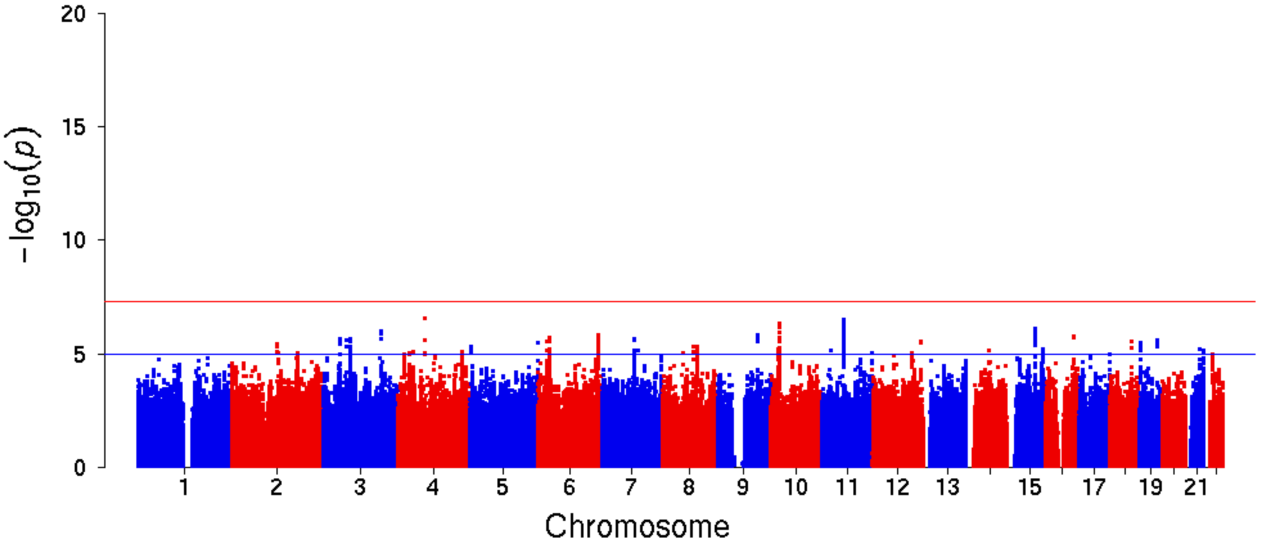

Supplementary Figure 15: **Manhattan plot of Vig-PA-Home time GWAS.** *P*-values were calculated using a linear regression model, two-sided tests. The red and blue horizontal lines indicate the genome-wide significance (*p*-value=5×10^{-8}) and suggestive significance (*p*-value=10^{-5}) levels, respectively.

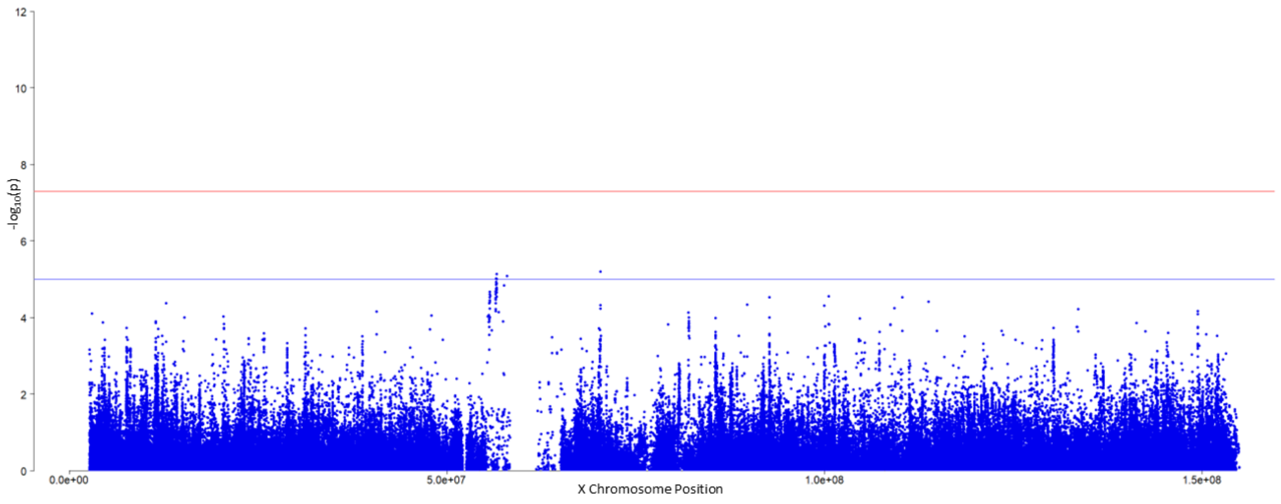

Supplementary Figure 16: **XWAS of Vig-PA-Leisure phenotype of EUR MVP data.** *P*-values were calculated using a linear regression model, two-sided tests. The red and blue horizontal lines indicate the genome-wide significance (*p*-value=5×10^{-8}) and suggestive significance (*p*-value=10^{-5}) levels, respectively.

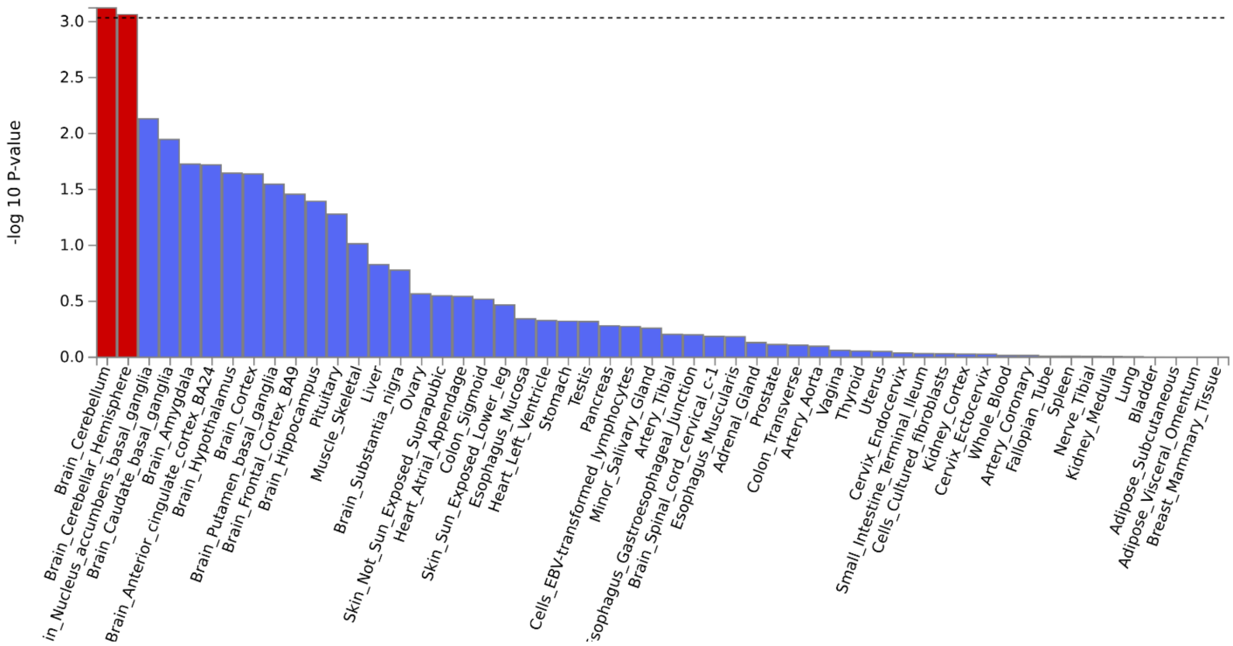

Supplementary Figure 17: **MAGMA Tissue Expression Analysis with specific tissue types for Vig-PA-Leisure GWAS.** Tests were two-sided.

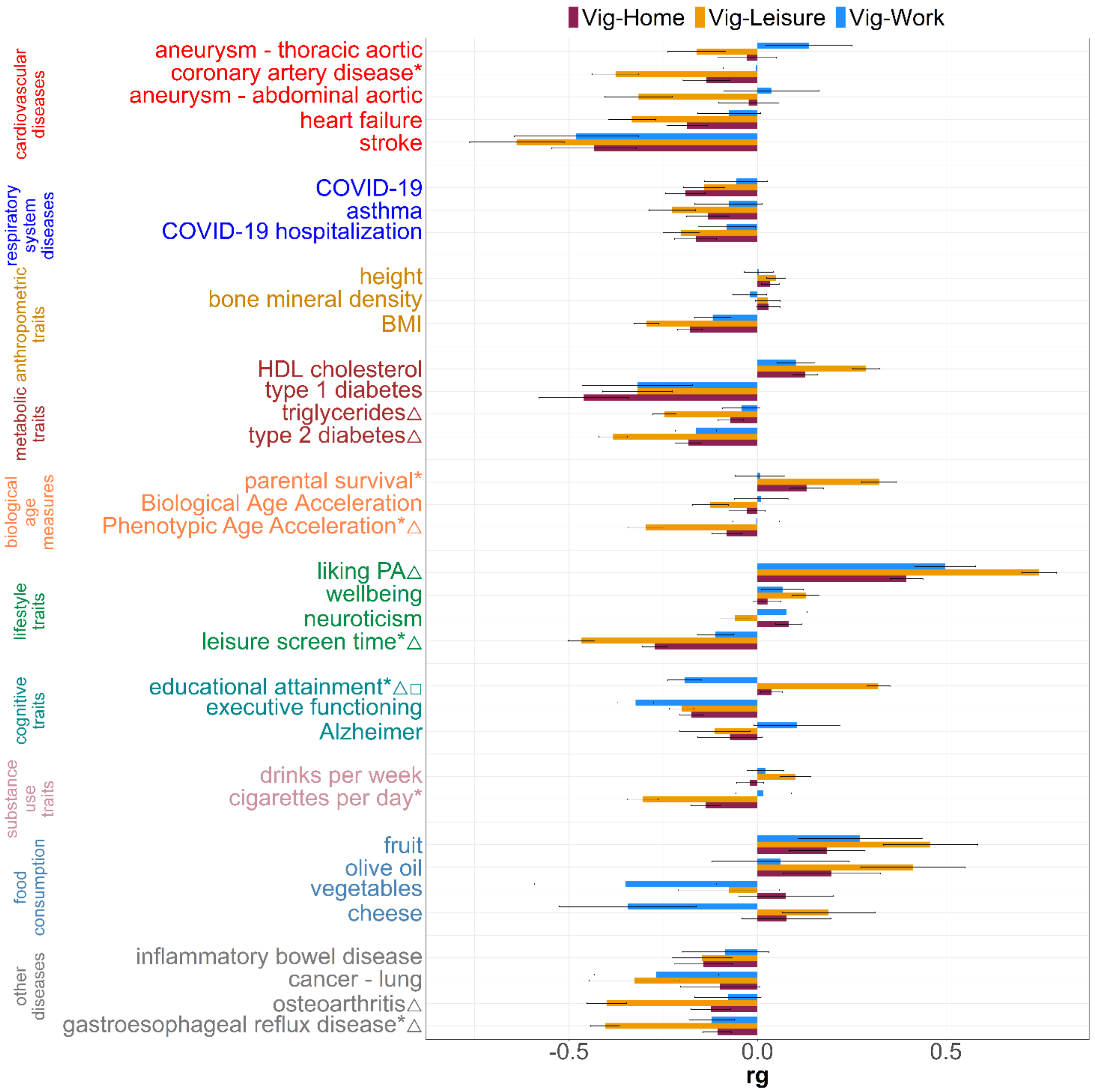

Supplementary Figure 18: **Genetic correlation between each pair of vigorous PA (Vig-PA-Leisure, Vig-PA-Work, Vig-PA-Home) after conditioning on income, and other traits of interest.** Significantly different correlations were defined having *p*-value < Bonferroni *p*-value threshold 4.76×10^{-4}: asterisk represents significant difference between Vig-PA-Leisure and Vig-PA-Work, triangle between Vig-PA-Leisure and Vig-PA-Home, square between Vig-PA-Home and Vig-PA-Work. Tests for genetic correlations were two-sided. Black error bars represent the standard error.

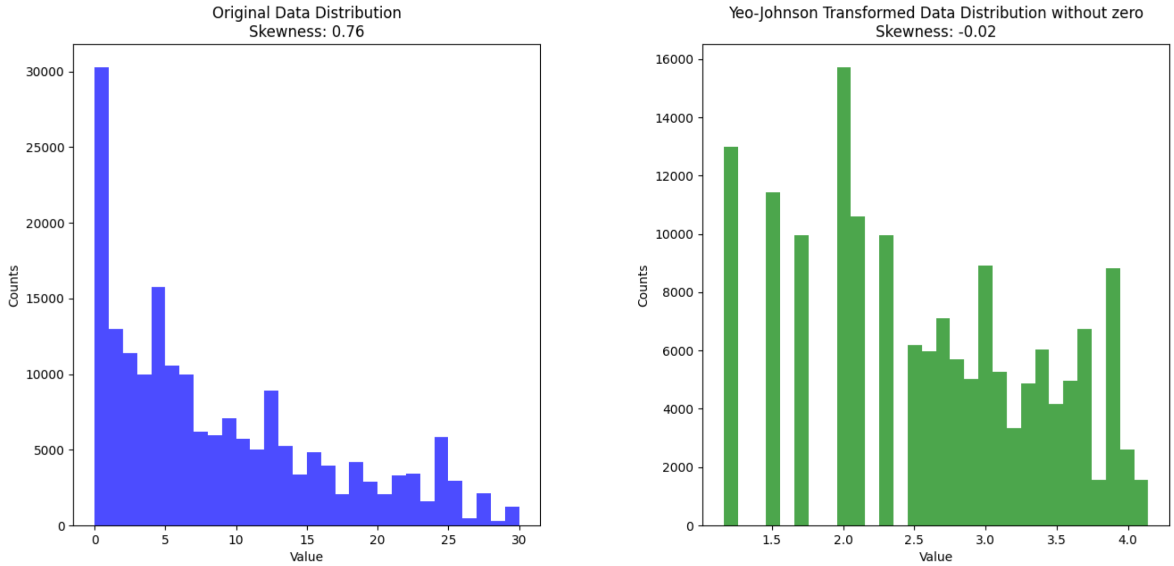

Supplementary Figure 19: **Distribution of All-PA-Leisure score and its Yeo-Johnson transformation for MVP EUR data.**

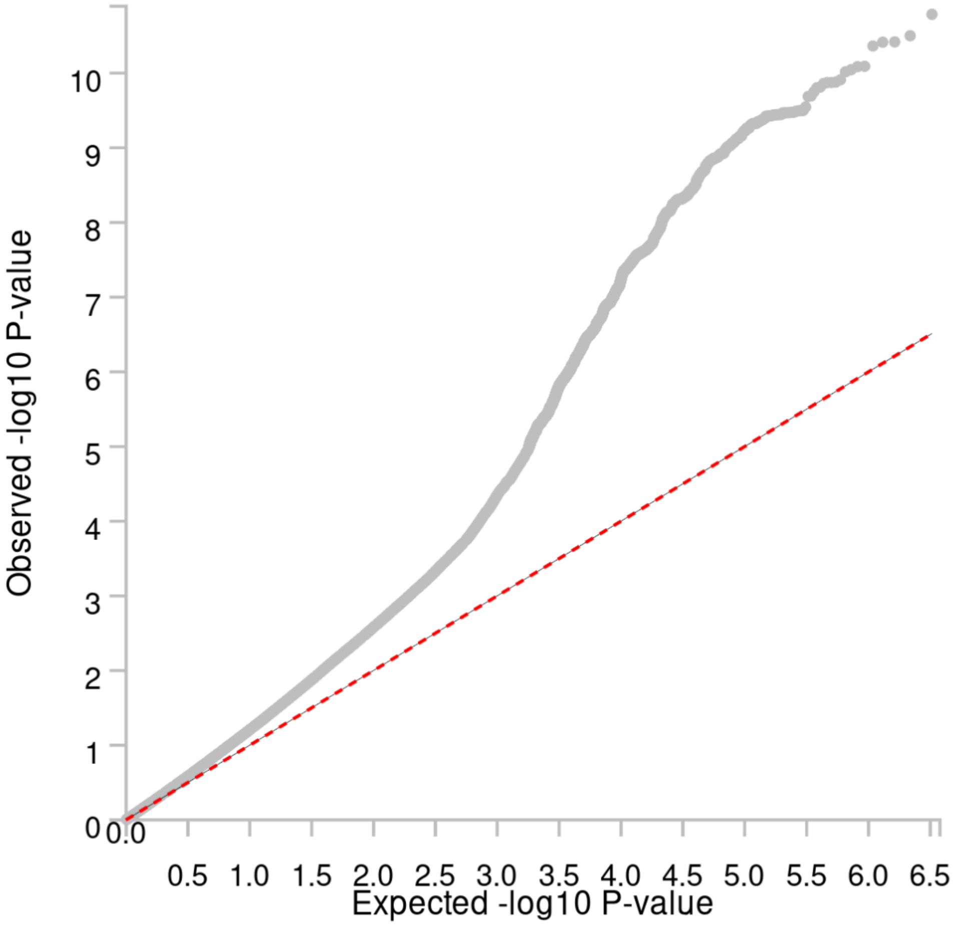

Supplementary Figure 20: **Quantile-quantile (QQ) plot, All-PA-Leisure phenotype in MVP EUR**. Intercept = 1.034±0.008; attenuation ratio of the LD score regression = 0.094±0.023. The observed *p*-values are plotted against their expected values under the null hypothesis assuming none of the sites have an effect.

VA Million Veteran Program core acknowledgement

VA Million Veteran Program:
Core Acknowledgements for Publications
May 2024

MVP Program Office

- Sumitra Muralidhar, Ph.D., Program Director
US Department of Veterans Affairs, 810 Vermont Avenue NW, Washington, DC 20420
- Jennifer Moser, Ph.D., Associate Director, Scientific Programs
US Department of Veterans Affairs, 810 Vermont Avenue NW, Washington, DC 20420
- Jennifer E. Deen, B.S., Associate Director, Cohort & Public Relations
US Department of Veterans Affairs, 810 Vermont Avenue NW, Washington, DC 20420

MVP Executive Committee

- Co-Chair: Philip S. Tsao, Ph.D.
VA Palo Alto Health Care System, 3801 Miranda Avenue, Palo Alto, CA 94304
- Co-Chair: Sumitra Muralidhar, Ph.D.
US Department of Veterans Affairs, 810 Vermont Avenue NW, Washington, DC 20420
- J. Michael Gaziano, M.D., M.P.H.
VA Boston Healthcare System, 150 S. Huntington Avenue, Boston, MA 02130
- Elizabeth Hauser, Ph.D.
Durham VA Medical Center, 508 Fulton Street, Durham, NC 27705
- Amy Kilbourne, Ph.D., M.P.H.
VA HSR&D, 2215 Fuller Road, Ann Arbor, MI 48105
- Michael Matheny, M.D., M.S., M.P.H.
VA Tennessee Valley Healthcare System, 1310 24th Ave. South, Nashville, TN 37212
- Dave Oslin, M.D.
Philadelphia VA Medical Center, 3900 Woodland Avenue, Philadelphia, PA 19104
- Deepak Voora, MD
Durham VA Medical Center, 508 Fulton Street, Durham, NC 27705

MVP Co-Principal Investigators

- J. Michael Gaziano, M.D., M.P.H.
VA Boston Healthcare System, 150 S. Huntington Avenue, Boston, MA 02130
- Philip S. Tsao, Ph.D.
VA Palo Alto Health Care System, 3801 Miranda Avenue, Palo Alto, CA 94304

MVP Core Operations

- Jessica V. Brewer, M.P.H., Director, MVP Cohort Operations
VA Boston Healthcare System, 150 S. Huntington Avenue, Boston, MA 02130
- Mary T. Brophy M.D., M.P.H., Director, VA Central Biorepository
VA Boston Healthcare System, 150 S. Huntington Avenue, Boston, MA 02130
- Kelly Cho, M.P.H, Ph.D., Director, MVP Phenomics
VA Boston Healthcare System, 150 S. Huntington Avenue, Boston, MA 02130
- Lori Churby, B.S., Director, MVP Regulatory Affairs
VA Palo Alto Health Care System, 3801 Miranda Avenue, Palo Alto, CA 94304
- Scott L. DuVall, Ph.D., Director, VA Informatics and Computing Infrastructure (VINCI)
VA Salt Lake City Health Care System, 500 Foothill Drive, Salt Lake City, UT 84148
- Saiju Pyarajan Ph.D., Director, Data and Computational Sciences
VA Boston Healthcare System, 150 S. Huntington Avenue, Boston, MA 02130
- Robert Ringer, Pharm.D., Director, VA Albuquerque Central Biorepository
New Mexico VA Health Care System, 1501 San Pedro Drive SE, Albuquerque, NM 87108
- Luis E. Selva, Ph.D., Director, MVP Biorepository Coordination
VA Boston Healthcare System, 150 S. Huntington Avenue, Boston, MA 02130
- Shahpoor (Alex) Shayan, M.S., Director, MVP PRE Informatics
VA Boston Healthcare System, 150 S. Huntington Avenue, Boston, MA 02130

- Brady Stephens, M.S., Principal Investigator, MVP Information Center
Canandaigua VA Medical Center, 400 Fort Hill Avenue, Canandaigua, NY 14424
- Stacey B. Whitbourne, Ph.D., Director, MVP Cohort Development and Management
VA Boston Healthcare System, 150 S. Huntington Avenue, Boston, MA 02130

MVP Publications and Presentations Committee

- Co-Chair: Themistocles L. Assimes, M.D., Ph. D
VA Palo Alto Health Care System, 3801 Miranda Avenue, Palo Alto, CA 94304
- Co-Chair: Adriana Hung, M.D.; M.P.H
VA Tennessee Valley Healthcare System, 1310 24th Ave. South, Nashville, TN 37212
- Co-Chair: Henry Kranzler, M.D.
Philadelphia VA Medical Center, 3900 Woodland Avenue, Philadelphia, PA 19104
