# Supplementary material for: A contextual genomic perspective on physical activity and its relationship to health, well being and illness
Source: Nat Genet. 2025 Jul 21;57(8):1860–71. doi: 10.1038/s41588-025-02260-9 (PMC12339386; doi:10.1038/s41588-025-02260-9)
Supplement: Supplementary file 2 — Reporting Summary [file 41588_2025_2260_MOESM2_ESM.pdf]

nature portfolio

Corresponding author(s): Joel Gelernter

Last updated by author(s): Jun 6, 2025

# Reporting Summary

Nature Portfolio wishes to improve the reproducibility of the work that we publish. This form provides structure for consistency and transparency in reporting. For further information on Nature Portfolio policies, see our Editorial Policies and the Editorial Policy Checklist.

## Statistics

For all statistical analyses, confirm that the following items are present in the figure legend, table legend, main text, or Methods section.

| n/a | Confirmed | |
|---|---|---|
| ☐ | ☒ | The exact sample size (*n*) for each experimental group/condition, given as a discrete number and unit of measurement |
| ☒ | ☐ | A statement on whether measurements were taken from distinct samples or whether the same sample was measured repeatedly |
| ☐ | ☒ | The statistical test(s) used AND whether they are one- or two-sided<br>*Only common tests should be described solely by name; describe more complex techniques in the Methods section.* |
| ☐ | ☒ | A description of all covariates tested |
| ☐ | ☒ | A description of any assumptions or corrections, such as tests of normality and adjustment for multiple comparisons |
| ☐ | ☒ | A full description of the statistical parameters including central tendency (e.g. means) or other basic estimates (e.g. regression coefficient) AND variation (e.g. standard deviation) or associated estimates of uncertainty (e.g. confidence intervals) |
| ☐ | ☒ | For null hypothesis testing, the test statistic (e.g. *F*, *t*, *r*) with confidence intervals, effect sizes, degrees of freedom and *P* value noted<br>*Give P values as exact values whenever suitable.* |
| ☒ | ☐ | For Bayesian analysis, information on the choice of priors and Markov chain Monte Carlo settings |
| ☒ | ☐ | For hierarchical and complex designs, identification of the appropriate level for tests and full reporting of outcomes |
| ☐ | ☒ | Estimates of effect sizes (e.g. Cohen's *d*, Pearson's *r*), indicating how they were calculated |

*Our web collection on statistics for biologists contains articles on many of the points above.*

## Software and code

Policy information about availability of computer code

| | |
|---|---|
| Data collection | n/a |
| Data analysis | We used the following software: PLINK 2.0 for GWAS, KING 2.0 for kinship, METAL (version March 3, 2011) for meta-analyses, XWAS 3.0 for X-wide association study, FUMA 1.5 for post-GWAS analyses, LDSC v1.0.1 for SNP-heritability and genetic correlations, LAVA 0.1.0 for local genetic correlations, TwoSampleMR v0.5.6 for Mendelian Randomization, FUSION (version May 24, 2022) for TWAS, FOCUS v0.7 for fine-mapping, MTAG 1.0.8 for multi-trait analysis of GWAS, g:profiler for enrichment analysis, R software SKAT v2.2.4 for mitochondrial gene-based association analyses, the R PheWAS package (March 31, 2023) for phenomewide association analysis, genomic-SEM v0.05 for genomic structural equation modeling, mtCOJO (from GCTA v 1.94.1) for multi-trait conditional and joint analyses, R package survival 3.3-1 for survival analysis. All software packages used in this analysis are publicly available. |

For manuscripts utilizing custom algorithms or software that are central to the research but not yet described in published literature, software must be made available to editors and reviewers. We strongly encourage code deposition in a community repository (e.g. GitHub). See the Nature Portfolio guidelines for submitting code & software for further information.

# Data

Policy information about availability of data

All manuscripts must include a data availability statement. This statement should provide the following information, where applicable:

- Accession codes, unique identifiers, or web links for publicly available datasets
- A description of any restrictions on data availability
- For clinical datasets or third party data, please ensure that the statement adheres to our policy

All MVP and meta-analysis summary statistics are made available through CIPHER https://phenomics.va.ornl.gov/web/cipher/partner/mvp: email CIPHER@va.gov with title "MVP and meta-analyses Summary Results – Galimberti et al".

# Research involving human participants, their data, or biological material

Policy information about studies with human participants or human data. See also policy information about sex, gender (identity/presentation), and sexual orientation and race, ethnicity and racism.

| | |
|---|---|
| Reporting on sex and gender | We have no available information on gender. Study design was population based and efforts were made to recruit both males and females. The MVP GWAS sample is mostly male (~91%), and sex was used as one of the covariates. |
| Reporting on race, ethnicity, or other socially relevant groupings | GWAS were conducted according to ancestry (European, African, Latin-American). |
| Population characteristics | The MVP includes veterans who have received care in the US VA Healthcare System. Participants were 65 years old on average. |
| Recruitment | For MVP recruitment, participants provided a blood sample for genomic analyses, granted access to medical records, and many agreed to complete two questionnaires, the MVP Baseline and Lifestyle Surveys.<br>UKB is a cohort study of ~500,000 adults that live in the United Kingdom (UK) and were recruited from 22 centers across the UK. |
| Ethics oversight | Research involving MVP in general is approved by the VA Central IRB, as for the current project. |

Note that full information on the approval of the study protocol must also be provided in the manuscript.

# Field-specific reporting

Please select the one below that is the best fit for your research. If you are not sure, read the appropriate sections before making your selection.

☒ Life sciences ☐ Behavioural & social sciences ☐ Ecological, evolutionary & environmental sciences

For a reference copy of the document with all sections, see nature.com/documents/nr-reporting-summary-flat.pdf

# Life sciences study design

All studies must disclose on these points even when the disclosure is negative.

| | |
|---|---|
| Sample size | Sample size reflected our best efforts to gather all possible participants with genetic data and available phenotypes as described. |
| Data exclusions | In MVP cohort, we excluded individuals without all the necessary information to run the GWAS analyses. |
| Replication | We ran independent GWAS of the two cohorts included in the meta-analysis and performed genome-wide genetic correlations. |
| Randomization | Observational study - not applicable |
| Blinding | Data were collected entirely independently of the analysts. There was no need for blinding or randomization. |

# Reporting for specific materials, systems and methods

We require information from authors about some types of materials, experimental systems and methods used in many studies. Here, indicate whether each material, system or method listed is relevant to your study. If you are not sure if a list item applies to your research, read the appropriate section before selecting a response.

## Materials & experimental systems

| n/a | Involved in the study |
|---|---|
| ☒ | ☐ Antibodies |
| ☒ | ☐ Eukaryotic cell lines |
| ☒ | ☐ Palaeontology and archaeology |
| ☒ | ☐ Animals and other organisms |
| ☒ | ☐ Clinical data |
| ☒ | ☐ Dual use research of concern |
| ☒ | ☐ Plants |

## Methods

| n/a | Involved in the study |
|---|---|
| ☒ | ☐ ChIP-seq |
| ☒ | ☐ Flow cytometry |
| ☒ | ☐ MRI-based neuroimaging |

# Plants

| | |
|---|---|
| Seed stocks | NA |
| Novel plant genotypes | NA |
| Authentication | NA |
